# ARTIST: High-Resolution Genome-Wide Assessment of Fitness Using Transposon-Insertion Sequencing

**DOI:** 10.1371/journal.pgen.1004782

**Published:** 2014-11-06

**Authors:** Justin R. Pritchard, Michael C. Chao, Sören Abel, Brigid M. Davis, Catherine Baranowski, Yanjia J. Zhang, Eric J. Rubin, Matthew K. Waldor

**Affiliations:** 1 Department of Microbiology, Harvard Medical School, Boston, Massachusetts, United States of America; 2 Department of Immunology and Infectious Disease, Harvard School of Public Health, Boston, Massachusetts, United States of America; 3 Division of Infectious Disease, Brigham and Women's Hospital, Boston, Massachusetts, United States of America; 4 Howard Hughes Medical Institute, Boston, Massachusetts, United States of America; University of Geneva Medical School, Switzerland

## Abstract

Transposon-insertion sequencing (TIS) is a powerful approach for deciphering genetic requirements for bacterial growth in different conditions, as it enables simultaneous genome-wide analysis of the fitness of thousands of mutants. However, current methods for comparative analysis of TIS data do not adjust for stochastic experimental variation between datasets and are limited to interrogation of annotated genomic elements. Here, we present ARTIST, an accessible TIS analysis pipeline for identifying essential regions that are required for growth under optimal conditions as well as conditionally essential loci that participate in survival only under specific conditions. ARTIST uses simulation-based normalization to model and compensate for experimental noise, and thereby enhances the statistical power in conditional TIS analyses. ARTIST also employs a novel adaptation of the hidden Markov model to generate statistically robust, high-resolution, annotation-independent maps of fitness-linked loci across the entire genome. Using ARTIST, we sensitively and comprehensively define *Mycobacterium tuberculosis* and *Vibrio cholerae* loci required for host infection while limiting inclusion of false positive loci. ARTIST is applicable to a broad range of organisms and will facilitate TIS-based dissection of pathways required for microbial growth and survival under a multitude of conditions.

## Author Summary

Transposon insertion sequencing (TIS) is a powerful method that couples high-density transposon mutagenesis with next-generation sequencing to comprehensively assess the fitness of thousands of transposon mutants across a genome. TIS is an extremely flexible technique that has been used to define genomic loci required for bacterial growth and survival in a variety of species and in many different growth conditions, including during host infection. However, there remain several important limitations to current TIS analysis methods. First, TIS data are not routinely normalized for the impact of experimental variability; second, most analyses are restricted to annotated loci and do not completely exploit the richness of TIS datasets; finally, TIS analysis methods are not easily accessible to most biologists. Here we present a pipeline—ARTIST—that addresses these issues and will transform TIS-based studies. We used ARTIST to conduct robust analyses of *Mycobacterium tuberculosis* and *Vibrio cholerae in vivo* TIS datasets and comprehensively defined the genetic requirements of these pathogens for host infection. The ARTIST pipeline will make TIS analysis accessible to many researchers and greatly enhance the rigor of and insights gained from TIS studies in a wide range of microorganisms.

## Introduction

Transposon-insertion sequencing (TIS) [1]–[4] is a powerful approach that enables rapid and comprehensive definition of an organism's genetic requirements for survival under a variety of different conditions (reviewed in [5], [6]). In TIS, a high-density transposon insertion library is grown under a condition of interest, and then subjected to high-throughput sequencing to map the transposon insertion site for each mutant in the library. The number of reads detected from each insertion mutant is proportional to the fitness of that mutant under the selected growth condition. Thus, strains carrying transposon insertions in loci required for survival will produce few or no reads, while reads from insertions that do not affect growth will be well-represented.

Genomic regions that are dispensable for growth in optimal laboratory conditions (e.g., rich media) but are required for survival in more stringent growth conditions are termed conditionally essential loci, whereas essential loci are thought to be required under all conditions. Because TIS is limited by sequencing capacity, here we use the terms ‘essential’ and ‘conditionally essential’ to define regions that are consistently underrepresented in reads in a given condition; the terms encompass both loci that are absolutely necessary for growth and those that can be disrupted, but are required for optimal growth. Identification of such regions, which can include non-coding sequences in addition to open reading frames, can yield considerable insight into the means by which an organism adapts to different environments. To date, comparative TIS-based studies have been carried out in diverse bacterial species and have defined genes required for survival in the presence of various nutrients and stresses as well as in experimental models of infection (reviewed in [5]).

While recent studies have firmly established the power and value of TIS, there are several shortcomings in current approaches used for TIS analysis that limit the optimal and widespread application of this technique for conditional essentiality screens. First, read counts between TIS libraries must be normalized to minimize the differences between libraries to be compared; however, current normalization protocols, which generally rely on scaling the frequency of all insertion mutants by a single factor to equalize the total number of reads per library [1], [7], do not take into account differences in library complexity that arise from stochastic processes encountered during the experiment such as biologic bottlenecks and sequence sampling error. Second, current methods for analysis of conditional essentiality are largely limited to annotated genomic features (e.g., ORFs, ncRNAs), which prevents *de novo* discovery of novel conditionally essential sequences. Finally, current TIS analysis methods usually rely on largely ad-hoc cutoffs (e.g., a minimum fold change in reads required for analytic consideration), and routinely utilize custom computational tools that are inaccessible to most biologists.

Here, we propose approaches to overcome these limitations of current conditional essentiality analysis. Our solutions include: 1) explicitly modeling changes in sequence abundance between libraries to simulate the systematic differences that can exist between TIS datasets; 2) a novel adaptation of a hidden Markov model, which enables annotation-independent prediction of functional importance across the entire genome; and 3) implementation of this pipeline on a single platform [8] using well-documented tools in order to offer biologists a standardized method of TIS analysis.

Our new analytic approaches are combined in a pipeline termed ARTIST (Analysis of high-Resolution Transposon-Insertion Sequences Technique), which includes both a previously characterized workflow for the definition of essential loci in a single TIS library [9] and a new pipeline for identification of conditionally essential loci. We validate the ARTIST pipeline by reanalyzing a TIS dataset from a recent study of *Mycobacterium tuberculosis* requirements for growth in a mouse model of infection [7]. Furthermore, we demonstrate ARTIST's versatility by carrying out a new analysis of *Vibrio cholerae* genomic requirements for infection in a rabbit model using a high-density transposon library created here. In both instances, ARTIST improves the detection of likely conditionally essential genes, while limiting false positive assignments. We believe the ARTIST pipeline will greatly facilitate and enhance the effectiveness of future TIS studies in a variety of organisms and conditions.

## Results and Discussion

### Overview of the ARTIST pipeline

**Figure 1:**
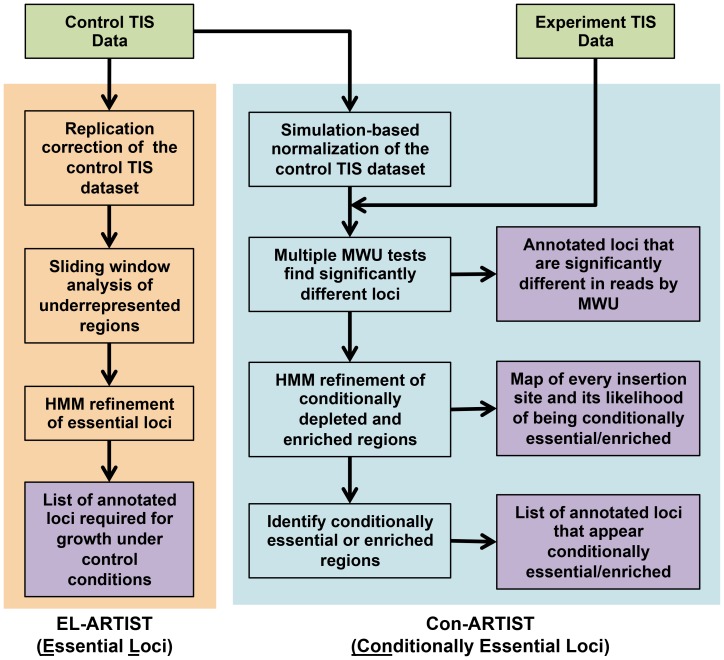
Flowchart of the ARTIST pipeline. Mapped reads from the control and experimental TIS libraries are tallied into read counts for every transposon insertion (see the Text S1 for example data formats). In the essential loci analysis arm, EL-ARTIST (shaded in orange), the control TIS data is first normalized to remove replication bias [34] and then a sliding window method [35] is used to initially define stretches of insertions that appear underrepresented in the data. These regions are subsequently used to train a hidden Markov model (HMM), which will refine the grouping of adjacent insertions into essential regions across the entire genome. The steps in this arm have been previously published [9]. In the conditionally essential analysis arm, Con-ARTIST (shaded in blue), the control dataset is first subjected to simulation-based normalization to compensate for genetic drift and sampling error. The resulting simulated control datasets are then compared to the experimental dataset using Mann-Whitney U (MWU) rank sum tests. Genes that are significantly different in reads with high reproducibility can be defined at this step. Alternatively, significantly under- and overrepresented genes from the MWU can serve as a training set for a HMM to further classify all genomic sites of insertion as conditionally depleted, enriched or unchanged between growth conditions. These assignments can be subsequently combined and used to categorize annotated genomic loci. Experimental data that is provided by the user are shown in green, while outputs from the ARTIST pipeline are shaded in purple.

The ARTIST pipeline is Matlab-based [8] and contains two different analysis tools (Figure 1). One arm, termed EL-ARTIST (for Essential Loci analysis), defines all loci that are required for growth (i.e., regions with few or no associated transposon insertions) in a TIS library generated under a single growth condition—commonly, standard laboratory conditions. While the key features of the EL-ARTIST analysis method were previously described [9], until now this approach was not publicly available as a standalone tool.

The second arm, Con-ARTIST (for Conditionally essential loci analysis) is a new tool that compares transposon libraries that have been grown under different conditions, in order to define conditionally essential loci that are only required for survival under a subset of growth conditions. The Con-ARTIST workflow includes two novel modules that improve upon current TIS analysis methods. First, simulation-based resampling aids normalization between libraries that have different frequencies of mutants due to stochastic experimental variation. Second, a hidden Markov model (HMM) dissects the genome in an annotation-independent manner, allowing the definition of both annotated and uncharacterized genomic regions according to their contribution towards growth.

In the first step of the Con-ARTIST workflow (Figure 1), mapped read counts from all transposon insertions are normalized between TIS datasets using simulation-based resampling of the control library. This creates independently simulated control libraries that reflect how mutant frequencies can change in an experiment simply due to chance events. For each of these simulated libraries, the number of reads within every annotated genomic feature (e.g., ORFs, ncRNAs, etc.) is compared to that of the same feature in the experimental dataset using a Mann-Whitney U (MWU) statistical test. Non-parametric statistical tests such as the MWU are preferred, as they make no assumptions about the distribution of reads in each dataset and thus may be less sensitive to biases in the experiment (e.g., PCR amplification jackpot events). These MWU tests identify annotated regions that contain significantly different numbers of reads in the control versus the experimental library. When MWU tests are performed on all simulated control datasets, they provide the user with an estimate of how significance values can change due to chance in the experiment.

The results from the MWU analyses can be used directly for hypothesis generation or to train an annotation-independent hidden Markov model. The HMM is a statistical model that decodes whether genomic regions belong to a particular biological category (e.g., required for growth *in vivo*) given the fold changes in read counts at every insertion site in the genome. The HMM output is a map of every potential transposon insertion site in the genome and each site's likelihood of being required or dispensable for growth under the experimental condition tested. As the HMM is annotation-independent, this allows the user to scan the genome at fine resolution (i.e., down to individual insertions) and discover novel loci that regulate growth, such as upstream regulatory elements in intergenic regions and domain-coding regions within annotated genes (see Text S1 for more details). Probabilities within annotated loci can also be combined and a general prediction of essentiality reported for every gene or genomic feature. The final output will be a table of all genomic loci and their predicted biologic states (e.g., no change in growth between experimental conditions, required for growth under the control condition, conditionally underrepresented or conditionally overrepresented). All ARTIST scripts and example files (Dataset S1) as well as a comprehensive user manual (see Text S1) are provided in the supplementary materials.

### Genetic drift and sampling error limit the accuracy of conditional essentiality TIS analysis

Deriving meaningful results from conditional TIS experiments relies on understanding whether differences in mutant frequencies between libraries arise from selection or chance. There are two principal processes that can cause stochastic differences in mutant abundance and interfere with downstream analytical accuracy in TIS-based genetic screens: genetic drift and sampling error. In the context of a population of transposon mutants, genetic drift can be thought of as change in mutant frequencies due to random events, such as population bottlenecks and expansions [10], [11]. A stringent bottleneck will markedly alter the complexity of a library independent of the fitness of its constituent mutants (Figure S1A,B) while sampling error occurs when low abundance mutants in a mixed population are missed solely due to low sequencing saturation (Figure S1C,D).

Comparative analyses of Himar transposon libraries created in *M. tuberculosis* (previously described by Zhang et al. [7]) and *V. cholerae* (constructed for this study) grown *in vitro* and in animal hosts provide clear evidence for the existence of host bottlenecks and for stochastic variability in library recovery (see below). For example, we created a *V. cholerae* library that was used to inoculate infant rabbits, a model host for the study of cholera [12]. This library contained transposon insertions in>60% of all possible insertion sites (i.e., TA dinucleotides), while mutant recovery was highly variable between individual animals, ranging from 4–48% of possible sites disrupted (Figure S2A,B). In contrast, the libraries recovered from all rabbits collectively contained insertions in 57% of TA sites when a saturating number of reads (>3×10^6^) was sequenced, indicating that not all loss of *V. cholerae* library complexity in the host was due to selection. Similarly, the *in vitro M. tuberculosis* library contained insertions in ∼62% of potential insertion sites (Figure S2C), but libraries recovered from individual infected animals only had insertions in 26–41% of potential sites (Figure S2D).

When a relatively small proportion of the input *V. cholerae* library was lost during *in vivo* growth, the number of reads per locus was extremely reproducible among output libraries (e.g., rabbits 2 and 4; R = 0.95), whereas inter-animal correlation was less robust when the recovered libraries were less complex, presumably due to stochastic bottleneck-dependent processes (Figure S2E). Thus, host bottlenecks, which are present in most experimental TIS infection models [10], [11], [13], can present a major challenge for accurately discerning conditionally essential loci, and represent a stringent test of our method's ability to normalize TIS data.

### Multinomial distribution-based simulations model stochastic drift in TIS libraries

Routinely, TIS datasets with differing library complexity are multiplicatively scaled to the same number of total reads using a single factor [1], [7], which presumes that there will be proportional retention of all neutral mutations in the library, when in fact stochastic events can cause these proportions to change markedly. To address this issue, we used a multinomial distribution to resample reads from the control data and simulate the effect that stochastic processes may exert on the experimental dataset. This simulation relies on the assumption that the observed frequencies of insertion mutants in the deeply sequenced control library approximate their true proportions in the population, such that we can use the control frequencies to define the probabilities of a multinomial distribution. Specifically, the multinomial distribution is scaled by a factor derived from the proportional difference in library complexity (i.e., number of unique sites disrupted) between the control and experimental datasets; this difference approximates the extent of genetic drift and sampling error in the experiment. Next, we use this multinomial distribution to simulate control datasets that have the same number of total reads as the experimental dataset, but have been subjected to a stochastic loss of library complexity that is similar to that experienced by the experimental library. The simulation is repeated to create independently simulated control libraries, and the variance between these libraries reflects the extent that noise from chance events can influence the validity of downstream statistical tests.

To assess whether simulation-based resampling enables more accurate downstream statistical analysis, we first tested the robustness of multinomial-based normalization when the *in vitro* grown *V. cholerae* library was subjected to increasingly severe simulated bottlenecks. Bottleneck-passaged libraries and the original TIS library were normalized either by multinomial-based resampling or simple multiplicative scaling of reads. The normalized libraries were then compared against the original *in vitro* TIS dataset using a Mann-Whitney U statistical test. In this test, no genes should appear significant since all libraries are derived from the same original source. In Figure S3A, we found that multinomial-based normalization (Resampling) produced dramatically (3–5 fold) fewer false positive gene assignments when compared to multiplicatively scaling (MS) at all bottleneck stringencies. Thus, a multinomial-based normalization approach is more robust at mitigating the effects of population constrictions (i.e., bottlenecks) than the standard approach of multiplicative scaling.

**Figure 2:**
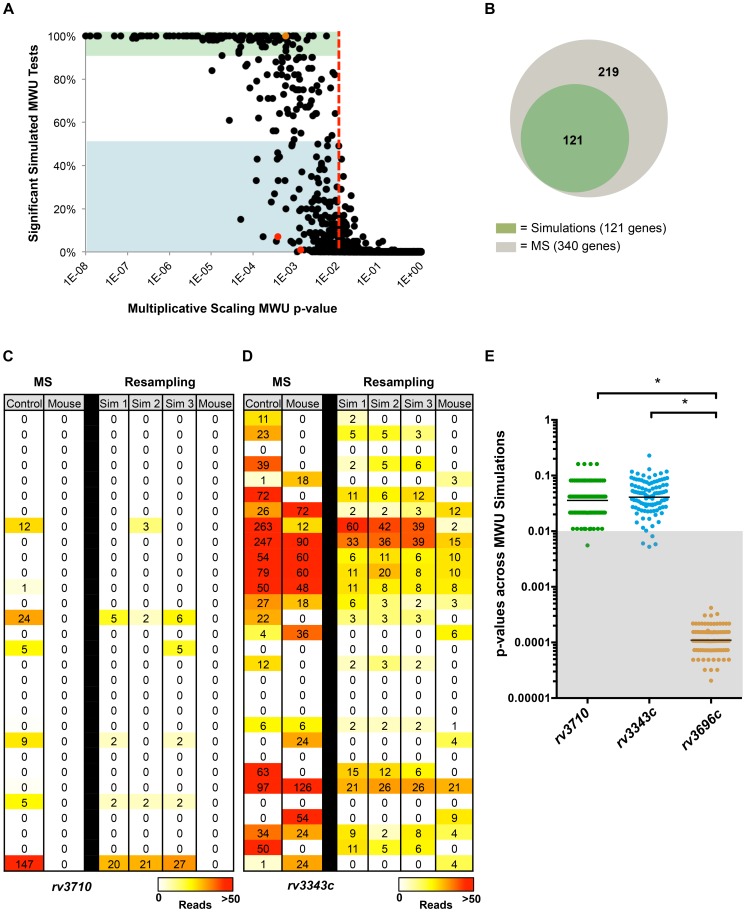
Simulation-based normalization allows identification of loci whose differential read abundance is unlikely to result from genetic drift or sampling error. (A) Two previously published *M. tuberculosis* datasets from transposon libraries grown *in vitro* and in a mouse infection model [7] were normalized to the same total reads using multiplicative scaling (MS), and then the genes in each library were compared with a Mann-Whitney U (MWU) statistical test. The same data was also subjected to simulation-based normalization, which models the effect of genetic drift and sampling error in the experiment. Simulation produced 100 new control libraries, which were then used to conduct 100 MWU tests against the mouse-passaged library. Each gene (dot) was then plotted by its MWU p-value derived from MS normalization alone, as well as its reproducibility in achieving a significant p-value (p<0.01, red line) across 100 MWU tests after simulation-based resampling. The blue shaded area contains genes that were significantly different in reads *in vivo* when MS normalization was used, but found to reach this level of significance less than 50% of the time upon simulation-based resampling. Green shaded genes were significant in over 90% of the resampling-based MWU tests. (B) Venn diagram showing the overlap between genes found using simulation-based normalization to have highly reproducible differences in read abundance (p-value<0.01 in over 90% of MWU tests; green) and genes found significant by MWU after multiplicative scaling (MS) only (gray). (C) Comparison of reads between *in vitro* and mouse grown *M. tuberculosis* libraries in a selected region of *rv3710* either after multiplicative scaling (MS) of the experimental library by a factor of 6 to achieve the same total reads between libraries, or after simulation-based normalization (resampling). Each row shows a potential insertion site (TA dinucleotide) in the gene and the number of reads detected at this site. The number of reads observed for each insertion is also depicted using a heat map. Data from three simulated control libraries is shown (Sim 1, Sim2, Sim3) to provide a sense of read variation between independent simulations. (D) Comparison of reads at a subset of insertion sites in *rv3343c* between *in vitro* and mouse grown *M. tuberculosis* libraries after multiplicative scaling or simulation-based resampling. (E) The p-values from 100 MWU tests performed after simulation-based normalization were plotted for the genes *rv3710*, *rv3343c* and *rv3696c*. The gray shaded area highlights MWU tests that yielded a significant result (p-value<0.01) when comparing reads between *in vitro* and *in vivo* libraries. The difference in distribution of p-values across MWU tests is significantly different between *rv3710* and *rv3343c* compared to *rv3696c* (* p-value<0.001).

Since we observed lower false positive rates in simulated data, we tested the merits of our multinomial normalization using animal infection data, which derives from a more complex and relevant biological system and thus offers a stringent test of our modeling approach. We re-analyzed previously published *in vitro* and mouse-grown *M. tuberculosis* datasets [7], and identified genes that were differentially represented *in vivo* (p-value<0.01) by MWU test after multiplicative scaling of libraries to the same total reads (as was performed by Zhang et al. but without applying their secondary read count threshold for defining significance). We also performed 100 simulations and MWU tests in the Con-ARTIST pipeline using the same data to model the effect of stochastic population changes in the experiment. Finally, we determined how reproducibly significant (p-value<0.01) the read count changes in all genes were across all 100 simulations, and compared these data to the multiplicatively scaled result above. While multiplicative scaling produced 340 genes with significant p-values, nearly 100 of those genes failed to reach the same level of significance in the majority of our simulations (Figure 2A, blue shaded area), suggesting that modeling stochastic mutant loss due to genetic drift may limit false positive assignments. Furthermore, only 121 genes were found to have significantly different read abundance (p<0.01) in over 90% of the simulation-based statistical tests (Figure 2A,B; green shaded areas). Importantly, all 121 genes would have also been predicted by multiplicative scaling, suggesting that we have not misidentified previously non-significant genes.

In-depth analyses of two of the strongest examples of irreproducibility—*rv3710* and *rv3343c* (Figure 2A, red dots)—illustrate how the discrepancy between genes with low reproducibility in our analyses but significant p-values by multiplicative scaling can arise. Despite a relative paucity of transposon insertions in *rv3710* in the *in vitro* library, when compared to the mouse-passaged library, which lacks reads in the entire locus, the gene is found to be conditionally essential by MWU testing when the control library is normalized solely by multiplicative scaling (Figure 2C). However, rare insertions often disappear in resampled controls, which makes the locus appear far less significantly different in the majority of 100 MWU tests (Figure 2E). Discrepancies between results from the two normalization approaches can also occur for loci that are well represented by insertions in both *in vitro* and *in vivo* libraries, as is the case for *rv3343c* (Figure 2D). This gene is deemed significantly underrepresented *in vivo* when using multiplicative scaling, despite a relatively minor difference in reads between conditions (∼2 fold), likely because the rank sum-based MWU test is prone toward significance with large numbers of datapoints. Since *rv3343c* contains over 160 potential insertion sites and is well disrupted, the locus may appear statistically different due to the large number of datapoints on which the MWU test is run. In contrast, when *rv3343c* is subjected to simulation-based normalization, the range in reads from the control library becomes narrower, and significant differences are only observed for a small subset of the MWU comparisons (Figure 2E). The observations with *rv3710* and *rv3343c* indicate that noise introduced by genetic drift can account for apparently significant differences in read abundance for loci that are both under-disrupted or well-disrupted in the control dataset.

Simulation can also enhance the significance value and improve identification of genes that are conditionally overrepresented. For example, *rv3696c*, an enriched gene (i.e., more reads *in vivo* than *in vitro*; Figure S3B), has a p-value of ∼6×10^−4^ in multiplicative scaling-based analyses (Figure 2A, orange dot), while simulation-based normalization yields an average p-value of ∼1.2×10^−4^, and yields a significant result in all 100 simulation-based MWU tests (Figure 2E).

These observations suggest that modeling the effects of genetic drift and sampling error biases inherent in TIS datasets can enhance the robustness of statistical analyses. In particular, simulation-based normalization appears to enable detection of reproducibly significant genes while avoiding many likely false positives. Importantly, we could not compensate for these biases by simply increasing the p-value stringency when using multiplicatively scaled data, as many moderately significant, but highly reproducible genes such as *rv3696c* would be lost alongside the potential false positives with broad variances in p-values (Figure S3C).

### Novel adaptation of a hidden Markov model for conditional TIS analysis

Most current statistical analyses of TIS data [1], [7] only analyze insertions within annotated genes and therefore yield relatively low genomic resolution while also omitting intergenic regions. To circumvent these limitations, we incorporated a hidden Markov model-based module into Con-ARTIST, which seeks to predict ‘hidden states’ (i.e., regions that belong to particular biological categories) by analyzing observed ‘emissions’ (i.e., reads from transposon insertions). In TIS analysis, the HMM utilizes read counts from each insertion and those of the immediately preceding insertion to assign hidden states, and thus leverages the information inherent in bacterial genome architecture without aggregating insertions or restricting analysis to previously annotated genomic features. Recently, HMM-based approaches were independently used by us and another group [9], [14] to analyze TIS data for the identification of genomic regions required for *in vitro* growth of *V. cholerae* and *M. tuberculosis*, respectively. However, these approaches were limited to analyzing a single TIS dataset, and a new HMM framework is required to assign additional biological categories in the context of comparative TIS studies.

In Con-ARTIST, after simulation-based normalization, we compare the reads and calculate the fold change at every potential insertion site between the *in vitro* (control) simulations and *in vivo* (experimental) dataset. Next, MWU tests are conducted as described above for all annotated loci, and the results (i.e., the genes having been defined as significantly under- and overrepresented *in vivo*) are used to train both the emission probabilities of fold changes at individual transposon sites and the transition probabilities between biological states. Emission and transition probabilities along with the observed fold changes are then used by the Viterbi algorithm to predict—in an annotation independent manner—whether each insertion site in the genome most likely belongs to one of 4 biological categories: 1) sites that are fully dispensable during both *in vitro* and *in vivo* growth; 2) regions that are essential in both conditions; 3) regions that are conditionally enriched (overrepresented) in the experimental library; or 4) regions that are conditionally essential (underrepresented) *in vivo*.

### Con-ARTIST analysis is resistant to false positive assignments

**Figure 3:**
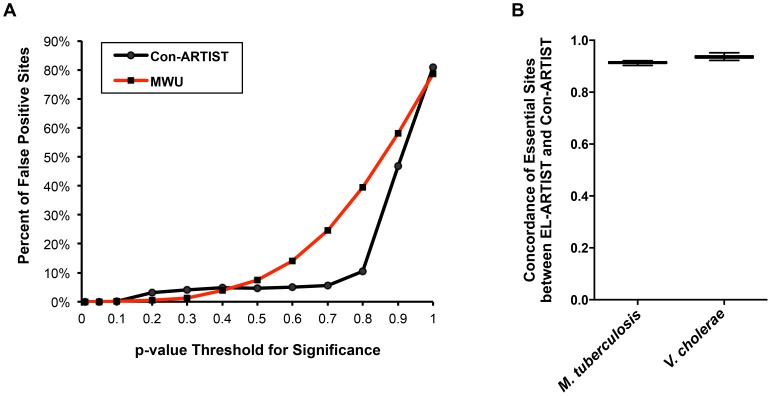
Con-ARTIST reduces false positive assignments. (A) Two simulated libraries derived from the same *M. tuberculosis* control dataset were compared against each other either using MWU analysis alone, or the full Con-ARTIST pipeline. We used a range of p-value cutoffs as the thresholds for defining whether an insertion site is being called significantly different in reads. The number of insertions that are called significantly different (i.e., being false positively assigned) between simulations when using a range of p-value cutoffs was then determined. (B) *In vitro* grown and animal infection datasets from *M. tuberculosis* and *V. cholerae* were run through the Con-ARTIST pipeline. The *in vitro* datasets from each organism were also analyzed by EL-ARTIST. The concordance of insertion sites similarly defined as essential for growth *in vitro* across 100 simulations was compared between the arms for both pathogens.

To demonstrate that our conditional HMM approach (Con-ARTIST) robustly assigns biological significance to different loci, we simulated several *in vitro* libraries of the Zhang et al. *M. tuberculosis* dataset [7] and then compared each of the simulated libraries against each other using either MWU analysis or Con-ARTIST (MWU followed by HMM). Because the simulated libraries are derived from the same dataset, a robust analysis method should not detect any significant differences between them. We used a range of p-value thresholds to define when loci were significantly different in reads, and determined the fraction of insertions that were thus false positively assigned. As expected from earlier simulations, both MWU and Con-ARTIST performed very well at low p-value cutoffs with virtually no insertions being called as significantly different in reads between simulations. However, Con-ARTIST had a more stable false positive rate across a wider dynamic range of p-values (>10% false positives at a p-value cutoff of 0.8) than the MWU method alone (>10% false positives at a p-value cutoff of 0.5), suggesting that the inclusion of the HMM is more resistant to false positive assignments than the MWU tests that it is trained upon (Figure 3A).

We further characterized the potential for false positives by comparing the *in vitro* essentiality assignments of each insertion site in *M. tuberculosis* and *V. cholerae* when the data are analyzed either by EL-ARTIST or Con-ARTIST. *In vitro* grown libraries of each organism were analyzed by EL-ARTIST and the essential and non-essential loci were defined, while Con-ARTIST was run on both the *in vitro* and *in vivo* grown libraries to define conditionally essential and enriched regions, in addition to essential and non-essential loci. Loci that are found to be required for *in vitro* growth should be highly concordant between EL-ARTIST and Con-ARTIST. Indeed, the agreement between essentiality assignments in *M. tuberculosis* and *V. cholerae* was approximately 91% and 95%, respectively, with little variation in 100 independent tests (Figure 3B), demonstrating that the inclusion of two additional biological categories (conditionally essential and enriched) to the Con-ARTIST HMM framework does not impact our ability to accurately define essential loci. Thus, Con-ARTIST appears robust relative to a HMM previously used for identification of essential loci [9].

### Con-ARTIST is more selective than previous analyses of conditionally essential loci

**Figure 4:**
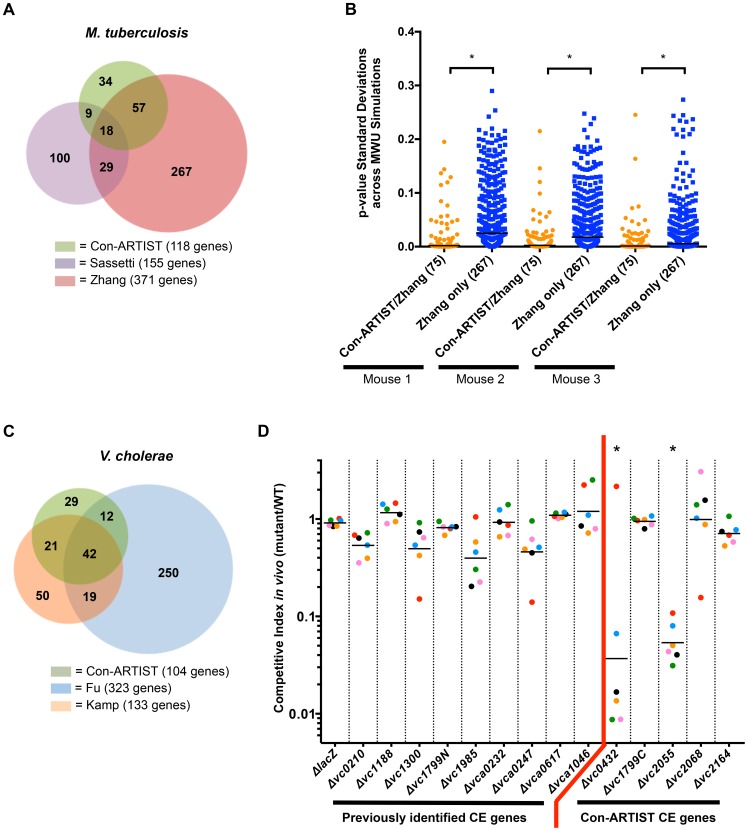
Con-ARTIST improves detection of conditionally essential genes compared to previous studies. (A) Overlap of the conditionally essential genes in *M. tuberculosis* required for mouse infection as determined by Con-ARTIST, Sassetti et al. [15] and Zhang et al. [7]. The overlap between Con-ARTIST and Sassetti et al. was significantly better than the overlap between Zhang et al. and Sassetti et al. (p-val<0.05, one-tailed Fisher's exact test). (B) The standard deviations of p-values across 100 simulation-based MWU tests were plotted for either Con-ARTIST conditionally essential genes in *M. tuberculosis* that overlap with Zhang et al. (75 genes), or for genes that were significant only in the Zhang et al. dataset (267 genes). In all three mice, genes that overlap between Con-ARTIST and Zhang et al. had significantly (*, p-value<0.0005) narrower ranges of p-value standard deviations across MWU tests than genes categorized as conditionally essential only by Zhang et al. (C) Overlap of conditionally essential genes required for *V. cholerae* rabbit infection as defined by Con-ARTIST, Kamp et al. [16] or Fu et al. [11]. Genes that were defined as defective for *in vitro* growth by Kamp et al. were filtered from both the Con-ARTIST and Fu et al. results. The overlap between Con-ARTIST and Kamp et al. was significantly higher than the overlap between Kamp et al. and Fu et al. (p-value<0.0001 by one-tailed Fisher's exact test). (D) In-frame deletions were constructed in several *V. cholerae* genes that were either identified as required for rabbit infection in previous studies (but not by Con-ARTIST) or unique predicted to be conditionally essential (CE) in this study. WT and mutant cells were first barcoded and then pooled to infect rabbits. The ratios of each mutant barcode compared to WT sequences were compared before and after infection to generate a competitive index. The competitive indexes for each mutant are coordinately colored according to the individual animal from which they were derived. One outlier measurement was identified (in *Δvc0432*) using the Grubbs test and removed. *, significantly underrepresented, p-value≤0.01 by Kruskal-Wallis test, with Dunn's test for multiple comparisons.

To further assess Con-ARTIST's utility, we compared *M. tuberculosis* and *V. cholerae* genes classified by Con-ARTIST as required for optimal *in vivo* growth to those identified in previously published TIS or microarray-based studies [7], [11], [15], [16]. For *M. tuberculosis*, the Con-ARTIST pipeline classified 118 genes (Table S1) as conditionally essential for mouse infection with high likelihood (all insertion sites within these genes have>90% probability of being conditionally essential. Most of these genes (84 or 71%) overlap with those identified in previous studies by Zhang et al. [7] or Sassetti et al. [15] (Figure 4A). This overlap is significantly greater (p-value<0.05 by one-sided Fisher's exact test) than the overlaps for the Sassetti et al. and Zhang et al. datasets, which were 36% and 28%, respectively. Additionally, in all three mice, the p-values for Con-ARTIST's conditionally essential genes that overlap with those of Zhang et al. had significantly lower standard deviations across the simulation-based MWU tests than did the p-values for genes that were identified only in Zhang et al. (Figure 4B), which includes a much larger set of conditionally essential genes (371), the majority of which were not found either by our analysis or the Sassetti et al. microarray study.

 Importantly, Con-ARTIST also identified genes known to be critical for *M. tuberculosis* virulence, such as members of the ESX-1 locus (*rv3865-rv3877*, Figure S4A), which encodes a virulence factor secretion system that is critical for pathogenesis and survival *in vivo* [17]. Notably, two conditionally essential (CE) genes known to be required for ESX-1 function, *rv3869* and *rv3871*, which encode a translocon subunit [18] and an ATPase [17], [19], respectively, were found to be required for infection by Con-ARTIST and Sassetti et al. [15], but not by Zhang et al. [7], indicating that Con-ARTIST is more sensitive than MWU tests alone in identifying conditionally essential genes when using the same raw data. Con-ARTIST also classified 34 genes as conditionally essential that were not identified in previous studies; many of these genes have consistently fewer read counts *in vivo* than *in vitro* (Figure S4B, Table S1), suggesting they may genuinely be important for infection.

In addition to re-analyzing published *M. tuberculosis* data, we constructed a new high-density transposon library in *V. cholerae*, and assessed which genomic regions were required for infection in an infant rabbit model of disease that closely mimics human cholera [12]. This model was recently used in two additional TIS–based studies [11], [16], and we compared those results to the output of Con-ARTIST. Con-ARTIST classified 201 genes as conditionally essential *in vivo* (Table S3); however, this list included genes whose disruption results in mild growth defects *in vitro* in a previous study [16]. Because such genes were treated separately by Kamp et al., we removed them from our output list and that of Fu et al. in order to facilitate accurate comparisons. Following this filtering step, 104 genes (Figure 4C) remained in Con-ARTIST's set of genes predicted (using probability cutoffs of 85%, 90%, or 95% produced the same results) to contribute specifically to growth *in vivo* (Figure S5A). The majority of the conditionally essential genes (72%) were also identified in at least one of the other two studies; in comparison, the overlaps for Kamp et al. and Fu et al. were 62% and 22%, respectively. Con-ARTIST's overlap with Kamp et al. is significantly higher (p-value<0.005 by one-sided Fisher's exact test) than the overlap between Kamp et al. and Fu et al. All three studies classified numerous genes known to be critical virulence factors as conditionally essential *in vivo* (Table S3), including proteins involved in the production of the type IV pilus, TCP, which mediates cell-to-cell adhesion [20] and is required for human infection [21]. Universally detected genes also included those encoding enzymes that mediate synthesis of various amino acids, suggesting that the host does not provide a sufficient supply of these nutrients to support *V. cholerae* growth in the small intestine (Figure S6).

Con-ARTIST did not identify 19 genes classed as conditionally essential by both previous analyses (Figure 4C). In our control library, these genes on average were disrupted at <50% of their TA sites, whereas almost all of the 104 conditionally essential genes that were identified by Con-ARTIST contained a significantly higher percentage of insertions (Figure S5B), suggesting that some conditionally essential genes may have been missed by our analysis because of low insertion frequencies in the control library rather than due to computational issues. We also constructed in-frame deletions for 8 genes that were defined as conditionally essential by Kamp et al. but were not found to be required by Con-ARTIST and tested the ability of these strains to colonize the rabbit host. None of the deletion strains had any apparent growth defect *in vitro* (Figure S5C) or significant attenuation in the host (Figure 4D). This result is consistent with our expectation that Con-ARTIST should reduce false positive assignments.

Con-ARTIST defined 29 *V. cholerae* conditionally essential genes that were not identified in either of the two previous studies. Many of these genes belong to pathways that have been previously implicated in *V. cholerae* growth *in vivo* [11], [16], suggesting that the Con-ARTIST classification is correct. For example, Con-ARTIST significantly (p-values<0.05 by one-sided Fisher's exact tests) defines more genes linked to oxidative phosphorylation and respiration as important for growth *in vivo* than found by Kamp et al. and Fu et al. (Figure S5D). We created deletions in 5 conditionally essential candidates that were defined solely by Con-ARTIST, and assessed the ability of these strains to colonize the rabbit. These mutants grew normally *in vitro* (Figure S5C), but two of the five mutants, *Δvc0432* and Δ*vc2055*, were significantly attenuated (p-value≤0.01) approximately 30-fold attenuated *in vivo* (Figure 4D). Thus, Con-ARTIST has the capacity to identify legitimate conditionally essential loci that were not found using other analysis methods, while apparently minimizing false positive calls, suggesting that the analysis pipeline is robust.

### Con-ARTIST enables high-resolution genomic interrogation of conditional essentiality

**Figure 5:**
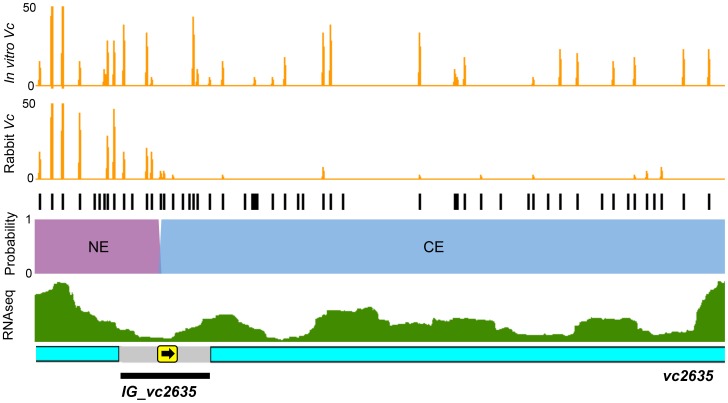
Con-ARTIST decodes intergenic regions of conditional essentiality. The *vc2635* locus, which encodes penicillin-binding protein 1A, was defined as conditionally essential *in vivo*, while an upstream intergenic region, *IG_vc2635*, was found to be domain conditionally essential by Con-ARTIST. The reads at each insertion in the *in vitro* and *in vivo* grown libraries are shown in orange and all potential transposon insertion sites (TA dinucleotides) are in black. The probabilities for each insertion being conditionally essential (CE, blue) or non-essential (NE, purple) *in vivo* (no probabilities for conditional enrichment or essential for *in vitro* growth were found) are overlaid on the locus. Previously published RNAseq data [23] was also overlaid on the locus (green). The part of the intergenic region identified as conditionally essential overlaps with the predicted −10 and −35 boxes (yellow box with arrow) and putative 5′ UTR sequence. The region presented shows reads spanning nucleotides 2805484 to 2806320 (∼800 bases).

Con-ARTIST enables annotation-independent identification of genomic regions of conditional essentiality, thereby facilitating definition of unannotated intergenic features, including non-coding RNAs (ncRNAs) and cis-acting regions. Con-ARTIST consistently classified 51 *V. cholerae* intergenic loci (Table S4) and 20 *M. tuberculosis* intergenic loci (Table S2) as conditionally essential or domain conditionally essential (i.e., containing both regions of conditional essentiality and regions that do not significantly vary in reads) *in vivo*. Over 90% of the *V. cholerae* intergenic regions were upstream of genes found to be required for host infection, suggesting they may identify promoters or 5′ UTRs that control the expression of downstream conditionally essential genes. An illustration of one such region—the intergenic region upstream of *vc2635*—is shown in Figure 5. *vc2635* encodes penicillin-binding protein 1A, a cell wall synthesis enzyme that was recently shown to be required for optimal *V. cholerae* growth *in vivo* [22]. Con-ARTIST defines the upstream intergenic region, *IG*_*vc2635*, as domain conditionally essential, and assigns the boundary between non-essential and conditionally essential sequence adjacent to the predicted −35 and −10 promoter sequences (Figure 5). Conditionally essential sequence extends uninterrupted from this site into the *vc2635* open read frame, which may suggest a polar effect of the transposon (e.g., disruption of a 5′ UTR or other regulatory region). Transcriptomic analysis of *V. cholerae* [23] detected transcripts that overlap well with the predicted conditionally essential region of IG_*vc2635* (Figure 5). This example highlights Con-ARTIST's utility in defining conditionally essential features within unannotated intergenic regions.

**Figure 6:**
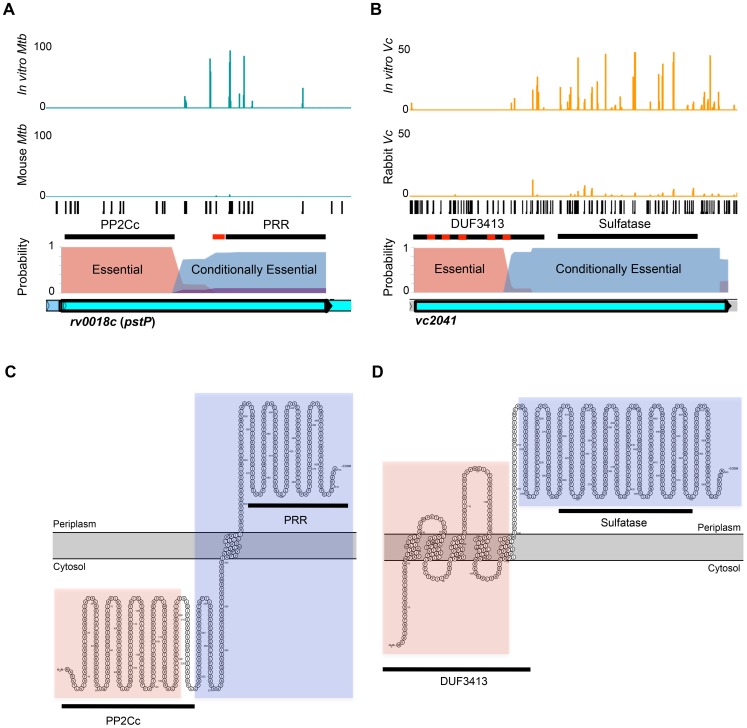
Con-ARTIST identifies conditionally essential regions required for host infection at sub-genic resolution. Con-ARTIST identified domain-encoding regions in *M. tuberculosis rv0018c* (A) and *V. cholerae vc2041* (B) that exhibit conditional essentiality *in vivo*. Reads in each organism (blue and orange) from *in vitro* and *in vivo* grown transposon libraries were mapped to each potential insertion site (TA dinucleotides, black bars) in the genome. Protein domains predicted by Pfam (black lines) and transmembrane segments predicted by Phobius (red boxes) are shown. The Con-ARTIST probabilities for each insertion being conditionally essential (blue), essential for growth *in vitro* (red) or non-essential *in vivo* (purple) are graphed along the gene (no probabilities for conditional enrichment were detected). The cellular compartments of Rv0018c (C) and VC2041 (D) protein domains were predicted and overlaid with the predicted Pfam protein domains (black lines) using Protter [36]. Con-ARTIST-defined regions of essentiality (pink shaded region) and conditionally essentiality (blue shaded region) were also overlaid. *rv0018c* covers ∼1.5 kb, while vc2041 is ∼1.8 kb.

Con-ARTIST can also define sub-genic regions of conditional essentiality, which can provide insight into gene domains that are important in particular environments. We identified 16 genes in *M. tuberculosis* and 22 genes in *V. cholerae* that appear to contain domains that are dispensable *in vitro* but required for *in vivo* growth (Table S1, S3). For example, *rv0018c*, which encodes the phosphatase PstP, tolerates insertions in its C-terminus-coding region *in vitro*, but these mutants were not recovered from infected mice (Figure 6A), suggesting that the product of this region is essential *in vivo*. In contrast, insertions within the N-terminus appear to prevent *M. tuberculosis* survival both *in vitro* and *in vivo*. Interestingly, the essential and conditionally essential regions correspond closely with predicted protein domains within the gene and their likely subcellular localizations: the essential serine/threonine phosphatase domain (PP2Cc) appears to be cytosolic, whereas the conditionally essential proline-rich region (PRR) is largely periplasmic (Figure 6C). Similarly, the uncharacterized *V. cholerae* gene *vc2041* is predicted by Con-ARTIST to consist of an N-terminal domain required for growth *in vitro* and a C-terminal region that is conditionally essential for survival in the rabbit (Figure 6B). The essential region covers a domain of unknown function (DUF3413) that contains several putative transmembrane passes, while the *in vivo* essential region overlaps a predicted sulfatase domain that is likely periplasmic (Figure 6D). Thus, Con-ARTIST appears capable of defining sequence encoding protein domains that likely have functionally distinct roles. The ability of Con-ARTIST to interrogate both intergenic and sub-genic regions in an annotation-independent manner across the entire genome is a novel and compelling aspect of this approach, as these features are typically omitted or missed by the aggregative statistical methods routinely used for conditional TIS analyses.

### Conclusions and perspectives

The ARTIST pipeline should be extremely useful for future genome-wide analyses of essential and conditionally essential loci in a variety of organisms. Users should note that while ARTIST takes certain TIS limitations into account, the analysis is most powerful when several TIS parameters are optimal: high starting library diversity, near sequencing saturation of every mutant and minimal impact from experimental bottlenecks. These metrics, however, are unique to different libraries, organisms and experimental setups. Consequently, we have provided scripts in the ARTIST package (Dataset S1) to assess these experimental parameters and enable users to determine whether ARTIST can provide an appropriate analysis for custom TIS data (see Text S1 for instructions and more in-depth discussion). We can report specifically from studies in *V. cholerae* and *M. tuberculosis* that we were able to generate accurate TIS analysis using Con-ARTIST when the starting TIS library contained insertions at>60% of all potential insertion sites, and that ∼50% of these insertions were retained after selection in the host. Also, unique mutant discovery in control and experimental libraries began to plateau at around 500,000 reads, and sequencing depths of 2–4 million reads typically produced 10–100 reads per neutral insertion, which was sufficient for Con-ARTIST analysis.

In addition, the HMM module of Con-ARTIST is designed for the analysis of sequentially derived interrelated libraries (i.e., all mutants recovered from one growth condition are a subset of the control library), and HMM analysis of libraries derived in parallel (i.e., libraries that may contain a different complement of insertion mutants) may be problematic. Nonetheless, analyses of individually derived libraries can still be conducted using Con-ARTIST without invoking the HMM module (see Text S1), albeit these analyses will be restricted to annotated loci rather than providing high-resolution insight at single insertion level.

Finally, although ARTIST was developed to analyze TIS datasets generated using Mariner-based transposons, which insert specifically at TA dinucleotides [24], [25], it should be adaptable to analysis of TIS data generated using Tn5 transposons, which have no absolute sequence specificity [4], [16], [26] (see Text S1 for details). We expect that with such modifications ARTIST will allow for annotation independent analysis of Tn5 data; however, its utility with Tn5-based TIS data has not yet been tested.

Despite these caveats, ARTIST represents a major step forward in making high-throughput TIS analysis publically available to biologists. ARTIST's novel normalization and high-resolution methodologies enable statistically rigorous annotation-independent identification of loci that are required for growth in one condition (EL-ARTIST) or loci that are conditionally essential or enriched in different conditions (Con-ARTIST), and will enhance the power of TIS-based studies in variety of organisms and experimental conditions.

## Materials and Methods

### Strains, media, and culture conditions

Wildtype *Vibrio cholerae* C6706 was grown on LB Miller (1% NaCl) supplemented with 200 ug/mL streptomycin (Sm), while the *V. cholerae* transposon mutant library was selected on LB+Sm+50 ug/mL kanamycin (Km). *Escherichia coli* SM10 lambda *pir* carrying the conjugative suicide transposon vector pSC189 [24] was grown in LB Miller supplemented with 100 ug/mL ampicillin. All *V. cholerae* and *E. coli* strains were grown overnight at 37°C unless otherwise indicated.

### *M. tuberculosis* TIS datasets

The *M. tuberculosis* data used in this study was taken from day 45, wildtype infected mice raw data from the Zhang et al. dataset [7].

### Construction of a high-density *Vibrio cholerae* transposon library

A single high-density transposon library was created in *V. cholerae* C6706 for rabbit infection studies. Briefly, pSC189 was conjugated into *V. cholerae* C6706 in 25 independent reactions. For each conjugation reaction, 200 ul of overnight stationary phase *E. coli* SM10 lambda pir carrying pSC189 and 200 ul of overnight stationary *V. cholerae* were washed in LB, mixed, pelleted, and finally resuspended in 100 ul of LB. The final resuspension was then spotted onto a 0.45 mm HA filter (Millipore, Billerica, MA, USA) on an LB plate and incubated at 37°C for 2 hours to allow conjugation (this typically yields ∼10000–30000 transposon mutants per conjugation). After conjugation, cells from the filter were resuspended in 1 mL LB by pipetting and vortexing in a conical tube. Resuspended cells from all filters were pooled and the final volume brought up to 30 mL with LB. Cells were then equally plated (3 mL) onto ten square 245×245 mm^2^ (Corning, Corning, NY, USA) LB+Sm+Km agar plates and grown at 30°C for 24 hours. In total, a single transposon library of ∼650000 colonies (containing insertions at 118683 TA dinucleotides, ∼62% of all potential insertion sites) was generated and used for downstream infection studies.

### Infection of infant rabbits with the *V. cholerae* transposon library

The animal protocols used for the studies described here were reviewed and approved by the Harvard Medical Area Standing Committee on Animals (Institutional Animal Care and Use Committee protocol number 04308, Animal Welfare Assurance of Compliance number A3431-01). All animal studies were carried out in accordance with the recommendations in the Guide for the Care and Use of Laboratory Animals of the National Institutes of Health (8th edition) and the Animal Welfare Act of the United States Department of Agriculture.

All transposon mutant colonies were resuspended on the selection plate with 10 mL of LB. The scraped cells were then pooled from all plates to create the final high-density *V. cholerae* transposon library. From this library, 3 mL of cells were pelleted, media removed, and stored at −80°C; this is the control transposon library. 1 mL of library cells was also pelleted, washed in sodium bicarbonate (2.5 g in 100 mL; pH 9) buffer and then further diluted in buffer to an OD_600_ of 1.4, which corresponds to ∼2×10^9^ CFU/mL. This is the inoculum for each rabbit. Infant rabbit infections were essentially performed as described previously [12]. In brief, 2–3 day old New Zealand White infant rabbits (Pine Acre Rabbitry, Norton, MA, USA) were treated with Zantac (ranitidine-hydrochloride; GlaxoSmithKline, Brentford, UK) by intraperitoneal injection (2 ug/g body weight). After 3 hours, each rabbit was intragastrically inoculated with 10^9^ CFU (500 ul) of the *V. cholerae* transposon library using a size 5 French catheter (Arrow International, Reading, PA, USA). The rabbits were housed with their mother and littermates for the duration of the experiment and euthanized at about 20 hours post-infection, or when severe watery diarrhea was observed.

At necropsy, the entire intestinal tract from the duodenum to the rectum was removed. A ∼5 cm long sample from the distal small intestine was collected, and homogenized in 1 mL sterile phosphate-buffered saline (PBS) using a mini-beadbeater-16 and two 3.2 mm stainless steel beads (BioSpec Products Inc., Bartlesville, OK, USA) for 2 minutes. The entire tissue homogenate was spread on a single 245×245 mm^2^ LB+Sm+Km plate. After growth for 18 hours at 30°C, the lawn of bacteria was washed off the plate with PBS, pelleted and stored at -80°C. Additionally, 1∶10 serial dilutions of the homogenates in PBS were plated for CFUs on LB+Sm+Km plates. The rabbits analyzed with the ARTIST pipeline (Rabbit 1, Rabbit 2 and Rabbit 4) had similar *V. cholerae* colonization (7.8×10^9^, 5×10^9^, 1.5×10^9^ CFUs/g tissue, respectively) to what was previously reported with this infection model [12].

### TIS library generation and sequencing

Genomic DNA extraction was performed on the *in vitro* and *in vivo* grown bacteria as previously described [9]. Briefly, each frozen pellet of bacteria was thawed, resuspended in 1 mL of dH_2_0 and then genomic DNA extracted using the Wizard Genomic DNA extraction kit (Promega, Madison, WI, USA). At least 10 volumes of all reagents were used for the lysis, protein removal and DNA precipitation steps for each pellet. Genomic DNA was spooled out of the final isopropanol DNA precipitation mixture using a blunted glass Pasteur pipette, successively washed in 70% and 100% ethanol and then air-dried at RT. The DNA was finally resuspended in ∼1–2 mL nuclease-free water at 65°C for at least 1 hour with frequent pipetting.

20 ug of genomic DNA was resuspended in 100 ul to a final concentration of 200 ng/ul, and then sheared to ∼200–600 bp fragments by sonication (Qsonica, Newtown, CT, USA) at 80% intensity, 30 s on, 30 s off for 40 minutes total at 4°C. After shearing, library preparation proceeded as previously described [7]. The frayed ends of 10 ug sheared DNA were repaired (Quick Blunt kit, NEB, Massachusetts, USA), adaptors ligated (sequences previously published [9]) and subjected to two rounds of PCR amplification to enrich for transposon insertion adjacent sequence and then to attach Illumina P5 and P7 hybridization sequence and barcodes for multiplexing. The final DNA libraries were gel purified on a 2% agarose gel and DNA fragments ranging from 300–500 bp extracted.

Sequencing of the libraries was conducted on a MiSeq (Illumina, San Diego, CA, USA) for 65 cycles, with a single read protocol. The resulting reads were trimmed for transposon and adaptor sequences using CLC Genomics Workbench (CLCbio, Aarhus, Denmark), and then mapped to the reference *V. cholerae* N16961 genome using the Bowtie aligner [27]. The total mapped reads for the *in vitro* input and three rabbit libraries were approximately 3×10^6^, 4×10^5^, 8×10^5^, and 1×10^6^, covering 118683, 50501, 85380, and 98546 insertion sites, respectively. This corresponds to disruption of 62%, 26%, 44% and 51% of all potential TA sites in these libraries. After mapping, the reads per TA site were tallied and assigned to annotated genes or intergenic regions using custom scripts (Dataset S1, Text S1). The raw read count data for all libraries can be found in Table S5.

### Construction and in vivo growth of *V. cholerae* deletion strains

Deletion plasmids for *V. cholerae* genes were constructed by ligating PCR products generated from the primers in Table S6 to the allelic exchange vector, pCVD442 [28] using Gibson assembly [29]. Sucrose-based counter-selection was performed as described previously [28], and the junctions of each in-frame deletion were confirmed using DNA sequencing. For *vc1799*, two internal deletions were created: an N terminal deletion of amino acids 5–299 (Δ*vc1799N*) and a C terminal deletion of amino acids 300–557 (Δ*vc1799C*). The deletion strains, along with WT and *lacZ*- *V. cholerae* were then barcoded at the intergenic region between *vc0610* and *vc0611* with unique 30 bp random sequence tags. The barcoding does not impact bacteria fitness *in vitro* or *in vivo* (Figure S5C), and each strain was independently tagged with distinct sequences. Tagged strains were grown and used for rabbit infections as described above. The total number of barcoded WT cells was approximately equal to the total number of mutant *V. cholerae* in the inoculum.

Rabbit tissue was harvested and *V. cholerae* gDNA isolated as described above. The sequence tags of all strains were amplified from the inoculum and rabbit samples using flanking primers and the PCR products subjected to Illumina sequencing (approximately 300,000 reads per sample). Low quality reads were discarded and extraneous sequences surrounding the barcode were trimmed using reaper-12-340 [30]. Sequences for each strain were clustered and read counts enumerated using uclust [31] from the QIIME 1.6.0 package [32] with a similarity threshold of 0.7. The frequency of each mutant in the rabbit samples relative to WT cells was then compared to the ratio of the mutants in the inoculum to generate a competitive index for each strain.

### The Con-ARTIST workflow

All ARTIST analyses were performed using custom scripts that are included as a single package in the supplementary materials alongside example data (Dataset S1). Detailed instructions on the system requirements and use of the ARTIST scripts are provided in the user manual, also in the supplementary materials (see Text S1).

Multinomial distribution-based random samplings were performed in Matlab using the mnrnd.m function. Raw read counts were then transformed to proportions by dividing raw reads by the total number of reads. The difference in TA sites disrupted between libraries is used to adjust the probabilities for the reads at each insertion site, and another state of TA loss is added to the multinomial probability vector. The number of samplings is proportional to the number of reads sequenced, and rescaled to match the reads of the experimental library. This resampling is packaged in the custom simulateequalsaturation.m function.

Mann Whitney U tests were performed in Matlab using the ranksum.m function. Custom code is included to rapidly run the ranksum function across annotated genomic features (runmwuallboots.m function). Following the creation of simulated control datasets, each simulation is compared to the experimental dataset using a MWU test, where significant results are set at a user-specified p-value cutoff. The MWU results are used to train a hidden Markov model using the Baum-Welch algorithm. Emissions are discretized fold change ratios, as well as separate emission flags for a number divided by zero. The Baum-Welch algorithm is implemented in Matlab to estimate the transition and emission probabilities for 4 biological states (enriched, conditionally essential, essential *in vitro*, and non-essential). After independently training the HMM for each simulation, we run the Viterbi algorithm to decode the identity of the 4 hidden states for every insertion site in the genome. Finally, following the running of the HMM on each of the simulations, we calculate the proportion of simulations predicting a particular hidden state at every single insertion site. Con-ARTIST analysis was performed between a single *in vitro* grown *V. cholerae* or *M. tuberculosis* dataset against each individual rabbit or mouse-passaged library. The final conditional essentiality assignment for each locus in *V. cholerae* and *M. tuberculosis* was the consensus assignment between the three independent *in vivo* Con-ARTIST comparisons.

### False positive simulations

To assess the stability of our adaptation of the HMM in Con-ARTIST, we compared control libraries of *M. tuberculosis* input data using either the MWU or Con-ARTIST analysis method. At each p-value cutoff, 10 different simulations were compared in a pairwise fashion, and the average proportion of insertion sites that were called significantly different was assessed.

Additionally, using a previously published method [9], we assessed the essential gene set in the control library of *M. tuberculosis* and *V. cholerae* in the EL-ARTIST pipeline. We ran the same data in the Con-ARTIST pipeline, which also includes an essential category. We then compared the essential or nonessential identity of each insertion site as defined by EL-ARTIST or Con-ARTIST, and assessed false positive assignments by counting TA sites that disagree in essentiality between the two methods.

### Data visualization

Transposon insertion read counts, Con-ARTIST probabilities and transcriptomic transcripts were overlaid on the *M. tuberculosis* and *V. cholerae* genomes and visualized using the Artemis genome browser [33].

## Supporting Information

Dataset S1 The ARTIST scripts and example file formats. All the custom scripts required to utilize the ARTIST pipeline are located in this folder. Also included is a subfolder that contains several input file examples that are compatible with the ARTIST pipeline.
(ZIP)

Figure S1 Stochastic changes in the composition of transposon mutant libraries can occur through genetic drift and sampling error. (A) The frequencies of individual transposon mutants within a library can vary extensively due to genetic drift (e.g., passage through bottlenecks). Mutants with low abundance (red) are more likely to have their frequencies change (or be lost entirely) solely by chance when the library is passed through a severe bottleneck in comparison with a mild bottleneck or lack of one altogether. (B) The effect of bottlenecks on library diversity was simulated for the *in vitro* grown *M. tuberculosis* transposon library using a multinomial distribution derived from the frequency of all insertion mutants in the library. (C) Sampling error occurs when transposon-adjacent DNA from insertion mutants of low abundance in the library is not sequenced solely due to chance. (D) *In vitro* grown *M. tuberculosis* TIS data was sampled at several read depths to demonstrate that sampling error is more likely to be introduced at low sequencing depth, and is much reduced near saturating sequencing depth, where few new insertion mutants are likely to be discovered.
(TIF)

Figure S2 Sensitivity analysis and sequencing saturation of *M. tuberculosis* and *V. cholerae* transposon libraries. (A) Sensitivity analysis of *V. cholerae in vitro* and pooled *in vivo* grown libraries was performed, where decreasing numbers of reads were randomly sampled from each library and the number of unique transposon insertions in those samplings were plotted to visualize the sequencing saturation level of each library. (B) The total number of unique transposon insertions isolated and sequenced mapped reads from an *in vitro* grown and several rabbit passaged *V. cholerae* transposon libraries was graphed. (C) Sensitivity analysis of *M. tuberculosis in vitro* and pooled *in vivo* grown libraries was performed as described above for *V. cholerae*. (D) The number of unique transposon insertions mutants and sequenced mapped reads isolated from *in vitro* grown and three mouse passaged *M. tuberculosis* libraries was graphed. (E) Total reads from every gene and intergenic region in *V. cholerae* were compared between three independent rabbit-passaged libraries and the correlation coefficient (R) was calculated for these pairwise comparisons.
(TIF)

Figure S3 Simulation-based normalization reduces false positive assignments and facilitates detection of enriched genes. (A) The *in vitro* grown *V. cholerae* dataset was subjected to different bottlenecks *in silico*, where increasing numbers of unique transposon mutants were lost by chance. The *in vitro* library was passed through each bottleneck several times to create 10 independently passaged libraries. The read counts of mutants that remain after the bottleneck were then normalized to the master *in vitro* library (total of 2 million reads) either using multinomial-based simulation (‘Resampling’) or simple multiplicative scaling (‘MS’). Each normalized library was compared to the original *in vitro* dataset using a Mann-Whitney U test, and genes that were found to be significantly different (p-value<0.001) were considered false positive gene assignments. (B) *rv3696c* was found to be significantly different in reads between *in vitro* and *in vivo* grown *M. tuberculosis* libraries when data is normalized by multiplicative scaling. This effect is more apparent in the difference in reads (and is more statistically significant) when the data is normalized by simulation-based resampling. Each row represents a potential insertion site (TA dinucleotide) in the gene and the number of reads detected at this site. The number of reads observed for each insertion is also depicted using a heat map. (C) *rv3696c* (orange dot) is moderately significant by MWU test after multiplicative scaling, but this significance is highly reproducible when data is normalized by simulation-based resampling. Importantly, increasing the p-value stringency cutoff in MWU tests using multiplicative scaling (green arrow) will not only remove like false positives that have low reproducibility of significance in MWU tests, but also genes like *rv3696c* that are consistently different in reads.
(TIF)

Figure S4 Con-ARTIST identifies conditionally essential genes in important pathways in *M. tuberculosis*. (A) Comparison of conditionally essentiality (CE) assignments of genes of the ESX-1 locus from three *M. tuberculosis* TIS analyses. NE = not essential for infection. (B) Two genes, *rv2966c* (566 basepairs in length) and *rv3684* (1040 basepairs), were predicted by Con-ARTIST to be conditionally essential during infection, but were not found in previous studies. Reads from insertions in the *in vitro* and mouse-passaged libraries are plotted in blue, while all potential insertion sites (TA dinucleotides) are shown in black. The Con-ARTIST probabilities for each insertion being predicted as conditionally essential (blue) or non-essential (purple) *in vivo* are overlaid (no probabilities for *in vitro* essentiality or conditional enrichment were detected).
(TIF)

Figure S5 Con-ARTIST identifies conditionally essential genes in important pathways in *V. cholerae*. (A) *In vitro* and rabbit-passaged *V. cholerae* TIS data was analyzed by Con-ARTIST for each individual rabbit. Insertions were defined as conditionally essential if their probability of being assigned to this category exceeded the stringency cutoffs tested—85%, 90% or 95%. Insertions in each gene were combined and genes were then defined as conditionally essential (CE), in which every insertion probability has exceeded the stringency cutoff or domain conditionally essential (DCE), in which there are insertions in the gene that both exceed and do not pass the desired cutoff. In all animals, the total number of genes with conditionally essential regions stays the same regardless of the probability cutoff, though at higher levels of stringency some genes switch from being designated entirely conditionally essential to domain conditionally essential. (B) There were 19 genes that were similarly defined as conditionally essential by Kamp et al. and Fu et al., but were not found by Con-ARTIST (Figure 4C). These genes had significantly fewer disrupted TA sites in the *in vitro* input library compared to the 104 Con-ARTIST defined conditionally essential genes (*, p-value<0.005). (C) 13 candidate conditionally essential genes were deleted in *V. cholerae*, barcoded with a unique tag at a neutral locus and grown in LB alongside a tagged WT strain. Growth was monitored with OD_600_ measurements at 15-minute intervals. None of the deletion strains had an appreciable growth defect *in vitro* compared a non-barcoded WT strain that was grown in parallel. (D) *V. cholerae* conditionally essential genes found by Con-ARTIST, Kamp et al. [16], and Fu et al. [11] were mapped onto the predicted KEGG respiration pathway. Though all three studies defined genes in this pathway as being required for growth *in vivo*, Con-ARTIST found significantly more members than previous studies (p-value<0.05 by Fisher's exact test). Genes were classified as conditionally essential (CE) or non-essential (NE) for growth *in vivo*. Genes that were defined as defective for optimal growth *in vitro* by Kamp et al. are marked as ‘Sick’, and genes that were not evaluated by previous studies were marked as not determined (ND). Genes that were found to be sick were not evaluated for conditional essentiality by Kamp et al., and are thus marked with ‘-’.
(TIF)

Figure S6 TIS studies highlight amino acid biosynthesis pathways required for *V. cholerae* growth in rabbits. Conditionally essential genes required for *V. cholerae* rabbit infection (but not for growth in rich media *in vitro*) that were identified by either Con-ARTIST, Kamp et al. [16] or Fu et al. [11] were mapped to the KEGG amino acid biosynthesis network map. Steps in amino acid biosynthesis catalyzed by genes that have homologues in *V. cholerae* are shown in green. Red arrows represent processes catalyzed by *V. cholerae* enzymes for which transposon mutants are underrepresented *in vivo*.
(TIF)

Table S1 The requirement of *M. tuberuclosis* genes for growth and survival in mice. All genes in *M. tuberculosis* were categorized by Con-ARTIST for their contribution towards growth in mice. Con-ARTIST characterized each gene with the following code: 1 = domain conditionally essential (DCE); 2 = fully conditionally essential (CE); 3 = domain enriched (D_Enr); 4 = fully enriched (Enr); 5 = no change between *in vitro* and *in vivo* growth. The consensus call was determined across all 3 mice and compared to the conditional essentiality (CE) assignments of Zhang et al. and Sassetti et al.
(XLSX)

Table S2 The requirement of *M. tuberculosis* intergenic regions for growth and survival in mice. All intergenic regions (Igs) in *M. tuberculosis* were categorized by Con-ARTIST for their contribution towards growth in mice. Con-ARTIST characterized each gene with the following code: 1 = domain conditionally essential (DCE); 2 = fully conditionally essential (CE); 3 = domain enriched (D_Enr); 4 = fully enriched (Enr); 5 = no change between *in vitro* and *in vivo* growth. The consensus call was determined across all 3 mice.
(XLSX)

Table S3 The requirement of *V. cholerae* genes for growth and survival in rabbits. All genes in *V. cholerae* were categorized by Con-ARTIST for their contribution towards growth in rabbits. Con-ARTIST characterized each gene with the following code: 1 = domain conditionally essential (DCE); 2 = fully conditionally essential (CE); 3 = domain enriched (D_Enr); 4 = fully enriched (Enr); 5 = no change between *in vitro* and *in vivo* growth. The consensus call was determined across all 3 rabbits and compared to the conditional essentiality (CE) assignments of Kamp et al. and Fu et al. Each gene was also compared with the *in vitro* growth defective (Sick) results from Kamp et al.
(XLSX)

Table S4 The requirement of *V. cholerae* intergenic regions for growth and survival in rabbits. All genes in *V. cholerae* were categorized by Con-ARTIST for their contribution towards growth in rabbits. Con-ARTIST characterized each gene with the following code: 1 = domain conditionally essential (DCE); 2 = fully conditionally essential (CE); 3 = domain enriched (D_Enr); 4 = fully enriched (Enr); 5 = no change between *in vitro* and *in vivo* growth. The total TA sites within each IG was also calculated. For conditionally essential (CE) IG loci, we determined whether these regions are found upstream of a CE gene.
(XLSX)

Table S5 Total read counts from *in vitro* and rabbit-passaged libraries for every potential insertion site in *V. cholerae*. The total reads counts from *in vitro* and rabbit-passaged *V. cholerae* TIS libraries were mapped to all TA dinucleotides in the genome and assigned to a specific locus.
(XLSX)

Table S6 Primer sequences for constructing in frame gene deletions in *V. cholerae*.
(XLSX)

Text S1 The ARTIST user manual. The user manual provides background knowledge on the ARTIST pipeline and detailed instructions to carry out transposon-insertion sequencing analysis using the ARTIST scripts.
(DOCX)

## References

[1] van Opijnen T, Bodi KL, Camilli A (2009) Tn-seq: high-throughput parallel sequencing for fitness and genetic interaction studies in microorganisms. Nat Methods 6: 767–772. 19767758 10.1038/nmeth.1377 PMC2957483

[2] Gawronski JD, Wong SM, Giannoukos G, Ward DV, Akerley BJ (2009) Tracking insertion mutants within libraries by deep sequencing and a genome-wide screen for Haemophilus genes required in the lung. Proc Natl Acad Sci U S A 106: 16422–16427. 19805314 10.1073/pnas.0906627106 PMC2752563

[3] Goodman AL, McNulty NP, Zhao Y, Leip D, Mitra RD, et al (2009) Identifying genetic determinants needed to establish a human gut symbiont in its habitat. Cell Host Microbe 6: 279–289. 19748469 10.1016/j.chom.2009.08.003 PMC2895552

[4] Langridge GC, Phan MD, Turner DJ, Perkins TT, Parts L, et al (2009) Simultaneous assay of every Salmonella Typhi gene using one million transposon mutants. Genome Res 19: 2308–2316. 19826075 10.1101/gr.097097.109 PMC2792183

[5] Barquist L, Boinett CJ, Cain AK (2013) Approaches to querying bacterial genomes with transposon-insertion sequencing. RNA Biol 10: 1161–1169. 23635712 10.4161/rna.24765 PMC3849164

[6] van Opijnen T, Camilli A (2013) Transposon insertion sequencing: a new tool for systems-level analysis of microorganisms. Nat Rev Microbiol 11: 435–442. 23712350 10.1038/nrmicro3033 PMC3842022

[7] Zhang YJ, Reddy MC, Ioerger TR, Rothchild AC, Dartois V, et al (2013) Tryptophan biosynthesis protects mycobacteria from CD4 T-cell-mediated killing. Cell 155: 1296–1308. 24315099 10.1016/j.cell.2013.10.045 PMC3902092

[8] (2013) MATLAB and Statistics Toolbox Release, R2013b. Natick, Massachusetts, United States: The MathWorks, Inc.

[9] Chao MC, Pritchard JR, Zhang YJ, Rubin EJ, Livny J, et al (2013) High-resolution definition of the Vibrio cholerae essential gene set with hidden Markov model-based analyses of transposon-insertion sequencing data. Nucleic Acids Res 41: 9033–9048. 23901011 10.1093/nar/gkt654 PMC3799429

[10] Troy EB, Lin T, Gao L, Lazinski DW, Camilli A, et al (2013) Understanding barriers to Borrelia burgdorferi dissemination during infection using massively parallel sequencing. Infect Immun 81: 2347–2357. 23608706 10.1128/IAI.00266-13 PMC3697624

[11] Fu Y, Waldor MK, Mekalanos JJ (2013) Tn-Seq analysis of vibrio cholerae intestinal colonization reveals a role for T6SS-mediated antibacterial activity in the host. Cell Host Microbe 14: 652–663. 24331463 10.1016/j.chom.2013.11.001 PMC3951154

[12] Ritchie JM, Rui H, Bronson RT, Waldor MK (2010) Back to the future: studying cholera pathogenesis using infant rabbits. MBio 1 1 1: e00047–10. 10.1128/mBio.00047-10 PMC2912669 20689747

[13] Skurnik D, Roux D, Aschard H, Cattoir V, Yoder-Himes D, et al (2013) A comprehensive analysis of in vitro and in vivo genetic fitness of Pseudomonas aeruginosa using high-throughput sequencing of transposon libraries. PLoS Pathog 9: e1003582. 24039572 10.1371/journal.ppat.1003582 PMC3764216

[14] DeJesus MA, Ioerger TR (2013) A Hidden Markov Model for identifying essential and growth-defect regions in bacterial genomes from transposon insertion sequencing data. BMC Bioinformatics 14: 303. 24103077 10.1186/1471-2105-14-303 PMC3854130

[15] Sassetti CM, Rubin EJ (2003) Genetic requirements for mycobacterial survival during infection. Proc Natl Acad Sci U S A 100: 12989–12994. 14569030 10.1073/pnas.2134250100 PMC240732

[16] Kamp HD, Patimalla-Dipali B, Lazinski DW, Wallace-Gadsden F, Camilli A (2013) Gene fitness landscapes of Vibrio cholerae at important stages of its life cycle. PLoS Pathog 9: e1003800. 24385900 10.1371/journal.ppat.1003800 PMC3873450

[17] Hsu T, Hingley-Wilson SM, Chen B, Chen M, Dai AZ, et al (2003) The primary mechanism of attenuation of bacillus Calmette-Guerin is a loss of secreted lytic function required for invasion of lung interstitial tissue. Proc Natl Acad Sci U S A 100: 12420–12425. 14557547 10.1073/pnas.1635213100 PMC218773

[18] Brodin P, Majlessi L, Marsollier L, de Jonge MI, Bottai D, et al (2006) Dissection of ESAT-6 system 1 of Mycobacterium tuberculosis and impact on immunogenicity and virulence. Infect Immun 74: 88–98. 16368961 10.1128/IAI.74.1.88-98.2006 PMC1346617

[19] Guinn KM, Hickey MJ, Mathur SK, Zakel KL, Grotzke JE, et al (2004) Individual RD1-region genes are required for export of ESAT-6/CFP-10 and for virulence of Mycobacterium tuberculosis. Mol Microbiol 51: 359–370. 14756778 10.1046/j.1365-2958.2003.03844.x PMC1458497

[20] Kirn TJ, Lafferty MJ, Sandoe CM, Taylor RK (2000) Delineation of pilin domains required for bacterial association into microcolonies and intestinal colonization by Vibrio cholerae. Mol Microbiol 35: 896–910. 10692166 10.1046/j.1365-2958.2000.01764.x

[21] Herrington DA, Hall RH, Losonsky G, Mekalanos JJ, Taylor RK, et al (1988) Toxin, toxin-coregulated pili, and the toxR regulon are essential for Vibrio cholerae pathogenesis in humans. J Exp Med 168: 1487–1492. 2902187 10.1084/jem.168.4.1487 PMC2189073

[22] Dorr T, Moll A, Chao MC, Cava F, Lam H, et al (2014) Differential Requirement for PBP1a and PBP1b in In Vivo and In Vitro Fitness of Vibrio cholerae. Infect Immun 82: 2115–2124. 24614657 10.1128/IAI.00012-14 PMC3993429

[23] Mandlik A, Livny J, Robins WP, Ritchie JM, Mekalanos JJ, et al (2011) RNA-Seq-based monitoring of infection-linked changes in Vibrio cholerae gene expression. Cell Host Microbe 10: 165–174. 21843873 10.1016/j.chom.2011.07.007 PMC3166260

[24] Chiang SL, Rubin EJ (2002) Construction of a mariner-based transposon for epitope-tagging and genomic targeting. Gene 296: 179–185. 12383515 10.1016/s0378-1119(02)00856-9

[25] Rubin EJ, Akerley BJ, Novik VN, Lampe DJ, Husson RN, et al (1999) In vivo transposition of mariner-based elements in enteric bacteria and mycobacteria. Proc Natl Acad Sci U S A 96: 1645–1650. 9990078 10.1073/pnas.96.4.1645 PMC15546

[26] Barquist L, Langridge GC, Turner DJ, Phan MD, Turner AK, et al (2013) A comparison of dense transposon insertion libraries in the Salmonella serovars Typhi and Typhimurium. Nucleic Acids Res 41: 4549–4564. 23470992 10.1093/nar/gkt148 PMC3632133

[27] Langmead B, Salzberg SL (2012) Fast gapped-read alignment with Bowtie 2. Nat Methods 9: 357–359. 22388286 10.1038/nmeth.1923 PMC3322381

[28] Donnenberg MS, Kaper JB (1991) Construction of an eae deletion mutant of enteropathogenic Escherichia coli by using a positive-selection suicide vector. Infect Immun 59: 4310–4317. 1937792 10.1128/iai.59.12.4310-4317.1991 PMC259042

[29] Gibson DG, Young L, Chuang RY, Venter JC, Hutchison CA 3rd, et al (2009) Enzymatic assembly of DNA molecules up to several hundred kilobases. Nat Methods 6: 343–345. 19363495 10.1038/nmeth.1318

[30] Davis MP, van Dongen S, Abreu-Goodger C, Bartonicek N, Enright AJ (2013) Kraken: a set of tools for quality control and analysis of high-throughput sequence data. Methods 63: 41–49. 23816787 10.1016/j.ymeth.2013.06.027 PMC3991327

[31] Edgar RC (2010) Search and clustering orders of magnitude faster than BLAST. Bioinformatics 26: 2460–2461. 20709691 10.1093/bioinformatics/btq461

[32] Caporaso JG, Kuczynski J, Stombaugh J, Bittinger K, Bushman FD, et al (2010) QIIME allows analysis of high-throughput community sequencing data. Nat Methods 7: 335–336. 20383131 10.1038/nmeth.f.303 PMC3156573

[33] Rutherford K, Parkhill J, Crook J, Horsnell T, Rice P, et al (2000) Artemis: sequence visualization and annotation. Bioinformatics 16: 944–945. 11120685 10.1093/bioinformatics/16.10.944

[34] Gallagher LA, Shendure J, Manoil C (2011) Genome-scale identification of resistance functions in Pseudomonas aeruginosa using Tn-seq. MBio 2: e00315–00310. 21253457 10.1128/mBio.00315-10 PMC3023915

[35] Zhang YJ, Ioerger TR, Huttenhower C, Long JE, Sassetti CM, et al (2012) Global assessment of genomic regions required for growth in Mycobacterium tuberculosis. PLoS Pathog 8: e1002946. 23028335 10.1371/journal.ppat.1002946 PMC3460630

[36] Omasits U, Ahrens CH, Muller S, Wollscheid B (2014) Protter: interactive protein feature visualization and integration with experimental proteomic data. Bioinformatics 30: 884–886. 24162465 10.1093/bioinformatics/btt607

