# Supplementary figures and images for: ARTIST: High-Resolution Genome-Wide Assessment of Fitness Using Transposon-Insertion Sequencing
Source: PLoS Genet. 2014 Nov 6;10(11):e1004782. doi: 10.1371/journal.pgen.1004782 (PMC4222735; doi:10.1371/journal.pgen.1004782)

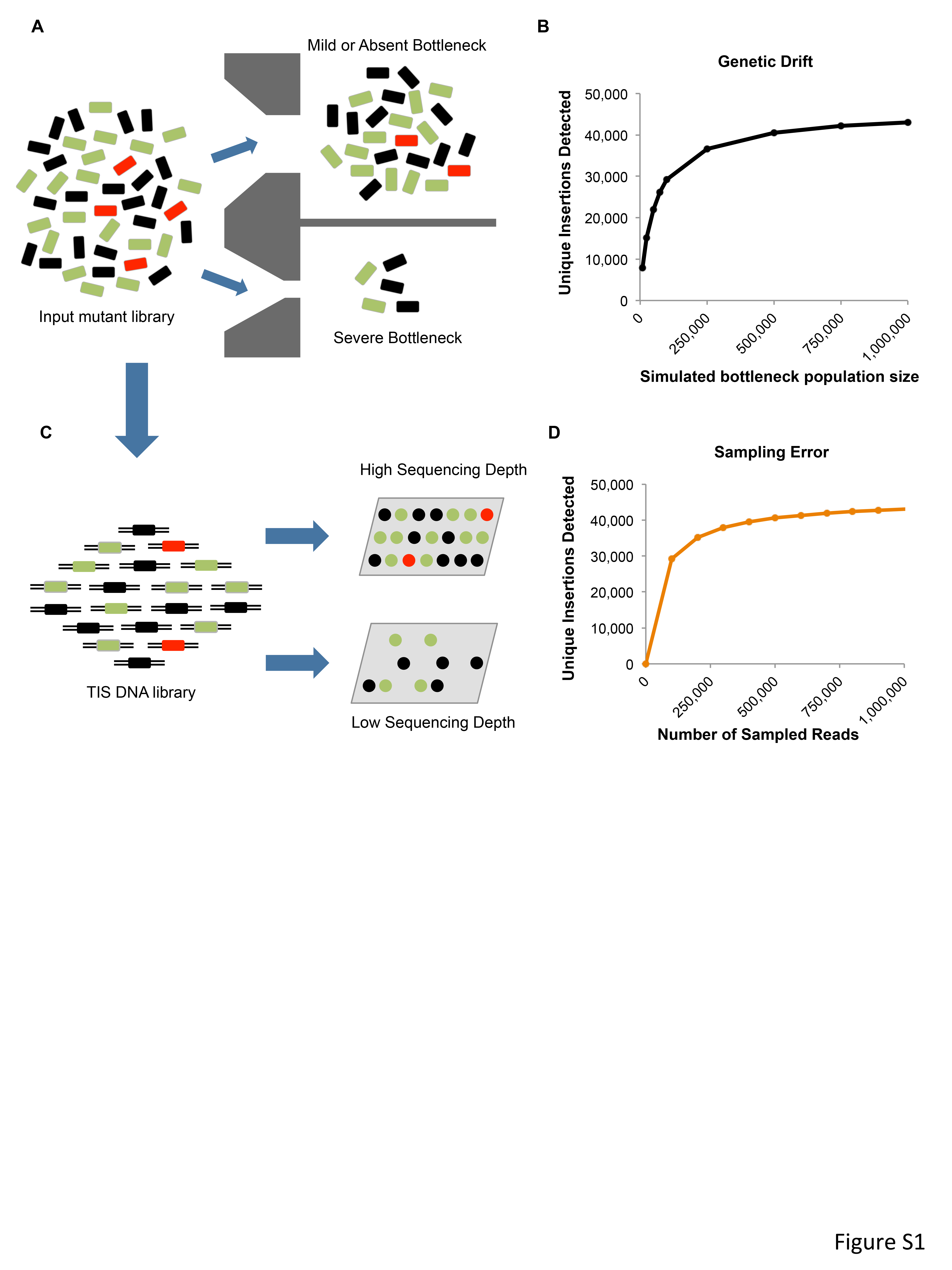

Supplement: Figure S1 — Stochastic changes in the composition of transposon mutant libraries can occur through genetic drift and sampling error. (A) The frequencies of individual transposon mutants within a library can vary extensively due to genetic drift (e.g., passage through bottlenecks). Mutants with low abundance (red) are more likely to have their frequencies change (or be lost entirely) solely by chance when the library is passed through a severe bottleneck in comparison with a mild bottleneck or lack of one altogether. (B) The effect of bottlenecks on library diversity was simulated for the in vitro grown M. tuberculosis transposon library using a multinomial distribution derived from the frequency of all insertion mutants in the library. (C) Sampling error occurs when transposon-adjacent DNA from insertion mutants of low abundance in the library is not sequenced solely due to chance. (D) In vitro grown M. tuberculosis TIS data was sampled at several read depths to demonstrate that sampling error is more likely to be introduced at low sequencing depth, and is much reduced near saturating sequencing depth, where few new insertion mutants are likely to be discovered. (TIF) [file pgen.1004782.s002.tif]

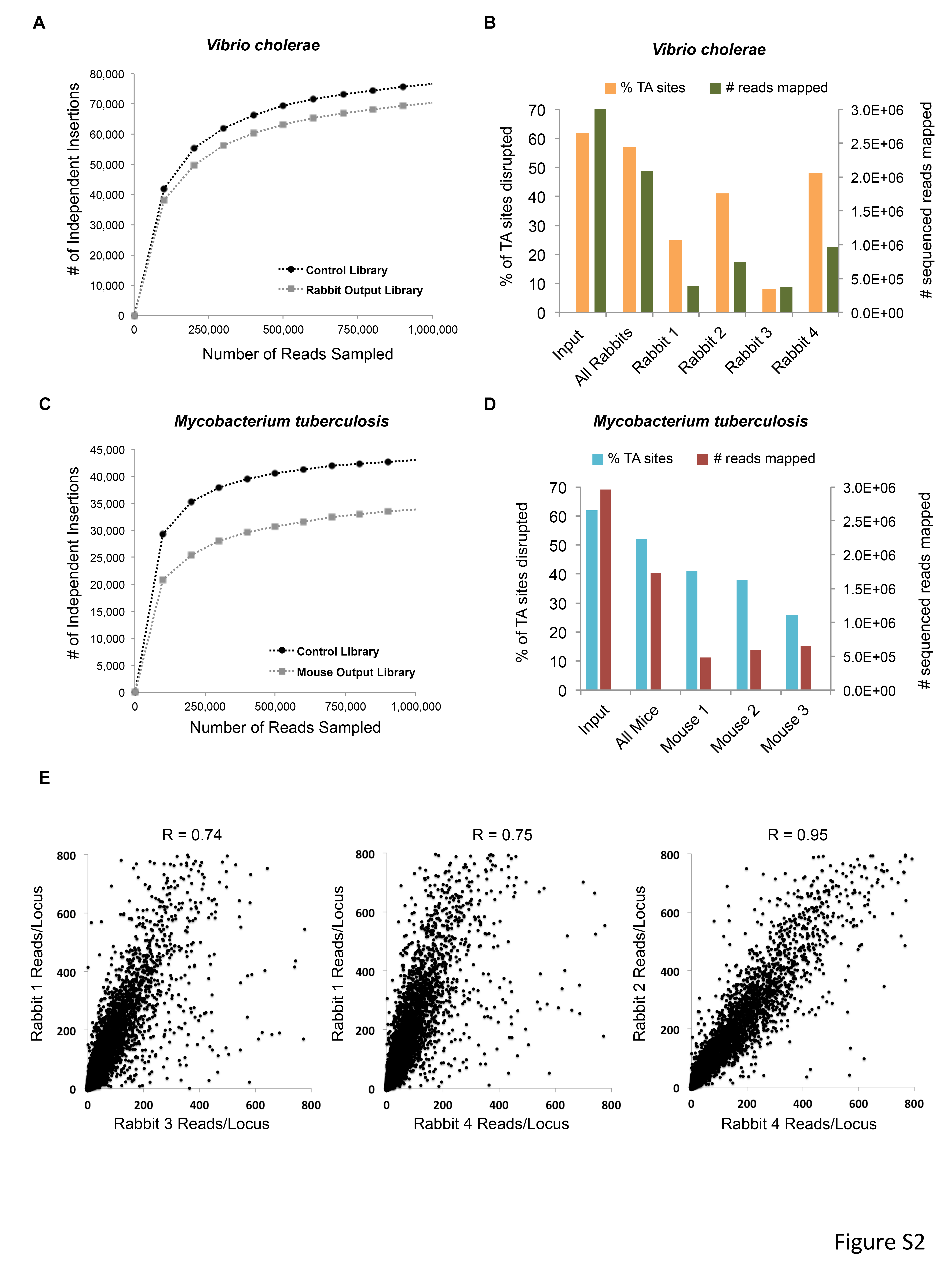

Supplement: Figure S2 — Sensitivity analysis and sequencing saturation of M. tuberculosis and V. cholerae transposon libraries. (A) Sensitivity analysis of V. cholerae in vitro and pooled in vivo grown libraries was performed, where decreasing numbers of reads were randomly sampled from each library and the number of unique transposon insertions in those samplings were plotted to visualize the sequencing saturation level of each library. (B) The total number of unique transposon insertions isolated and sequenced mapped reads from an in vitro grown and several rabbit passaged V. cholerae transposon libraries was graphed. (C) Sensitivity analysis of M. tuberculosis in vitro and pooled in vivo grown libraries was performed as described above for V. cholerae. (D) The number of unique transposon insertions mutants and sequenced mapped reads isolated from in vitro grown and three mouse passaged M. tuberculosis libraries was graphed. (E) Total reads from every gene and intergenic region in V. cholerae were compared between three independent rabbit-passaged libraries and the correlation coefficient (R) was calculated for these pairwise comparisons. (TIF) [file pgen.1004782.s003.tif]

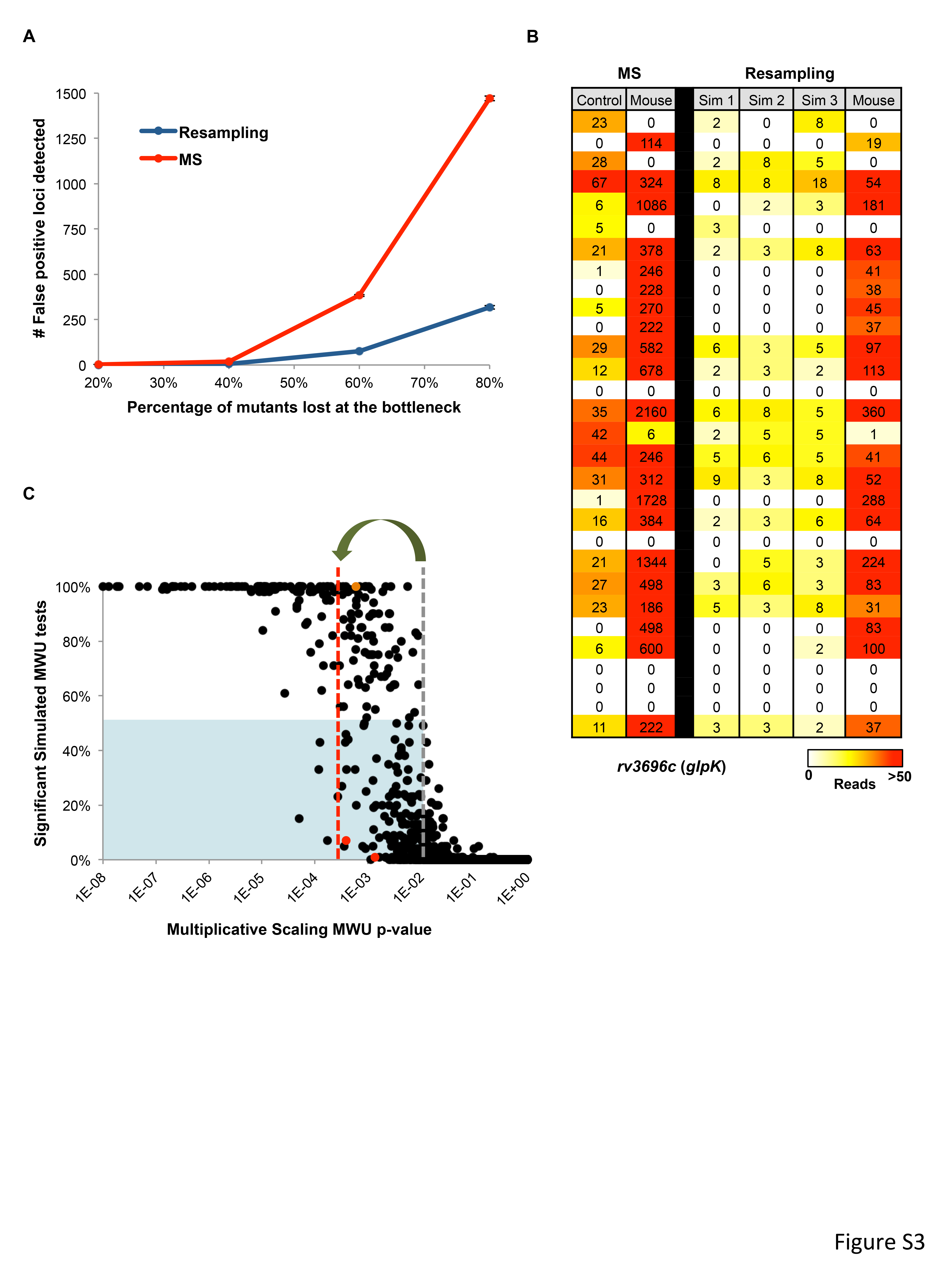

Supplement: Figure S3 — Simulation-based normalization reduces false positive assignments and facilitates detection of enriched genes. (A) The in vitro grown V. cholerae dataset was subjected to different bottlenecks in silico, where increasing numbers of unique transposon mutants were lost by chance. The in vitro library was passed through each bottleneck several times to create 10 independently passaged libraries. The read counts of mutants that remain after the bottleneck were then normalized to the master in vitro library (total of 2 million reads) either using multinomial-based simulation (‘Resampling’) or simple multiplicative scaling (‘MS’). Each normalized library was compared to the original in vitro dataset using a Mann-Whitney U test, and genes that were found to be significantly different (p-value<0.001) were considered false positive gene assignments. (B) rv3696c was found to be significantly different in reads between in vitro and in vivo grown M. tuberculosis libraries when data is normalized by multiplicative scaling. This effect is more apparent in the difference in reads (and is more statistically significant) when the data is normalized by simulation-based resampling. Each row represents a potential insertion site (TA dinucleotide) in the gene and the number of reads detected at this site. The number of reads observed for each insertion is also depicted using a heat map. (C) rv3696c (orange dot) is moderately significant by MWU test after multiplicative scaling, but this significance is highly reproducible when data is normalized by simulation-based resampling. Importantly, increasing the p-value stringency cutoff in MWU tests using multiplicative scaling (green arrow) will not only remove like false positives that have low reproducibility of significance in MWU tests, but also genes like rv3696c that are consistently different in reads. (TIF) [file pgen.1004782.s004.tif]

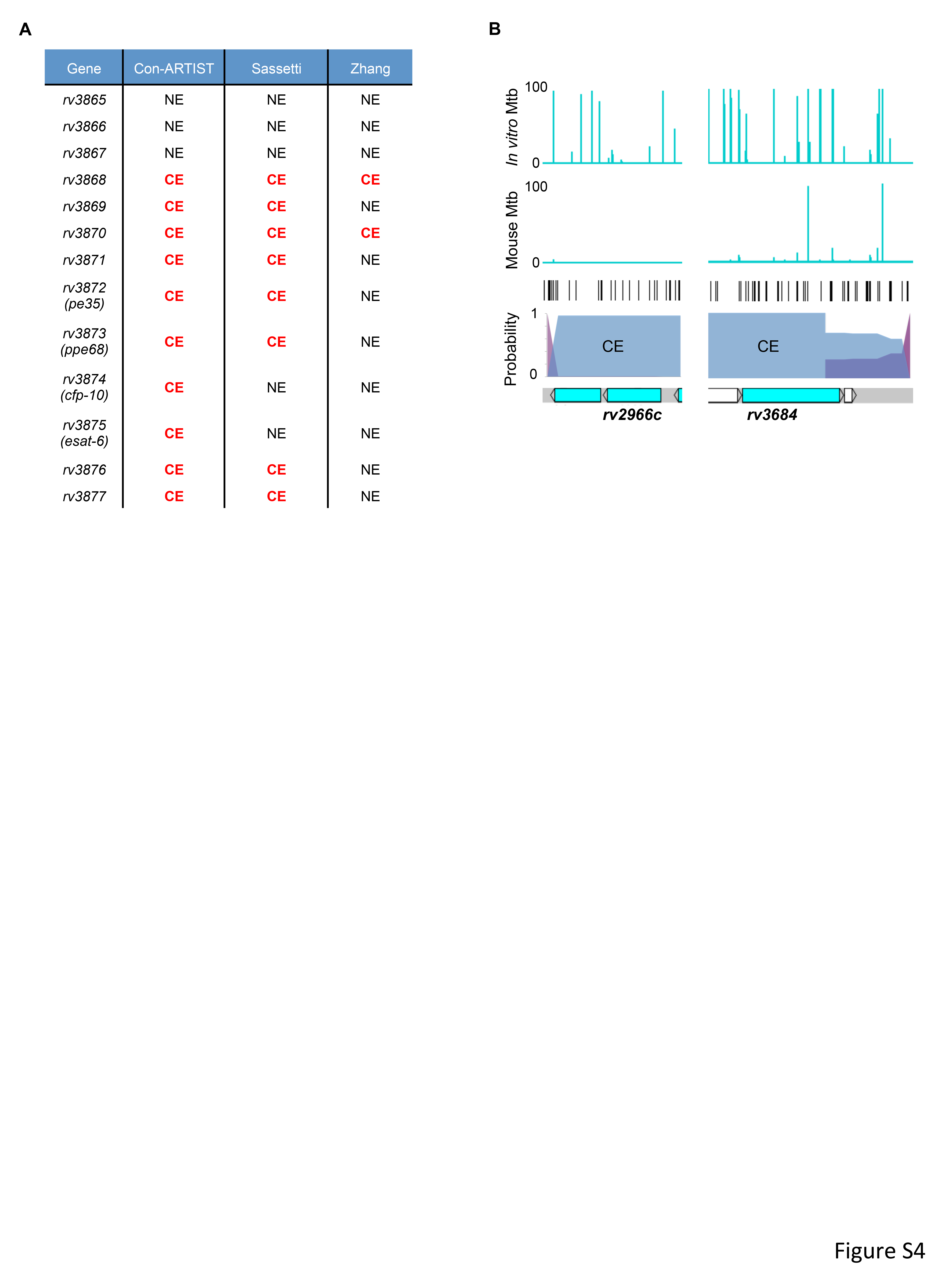

Supplement: Figure S4 — Con-ARTIST identifies conditionally essential genes in important pathways in M. tuberculosis. (A) Comparison of conditionally essentiality (CE) assignments of genes of the ESX-1 locus from three M. tuberculosis TIS analyses. NE = not essential for infection. (B) Two genes, rv2966c (566 basepairs in length) and rv3684 (1040 basepairs), were predicted by Con-ARTIST to be conditionally essential during infection, but were not found in previous studies. Reads from insertions in the in vitro and mouse-passaged libraries are plotted in blue, while all potential insertion sites (TA dinucleotides) are shown in black. The Con-ARTIST probabilities for each insertion being predicted as conditionally essential (blue) or non-essential (purple) in vivo are overlaid (no probabilities for in vitro essentiality or conditional enrichment were detected). (TIF) [file pgen.1004782.s005.tif]

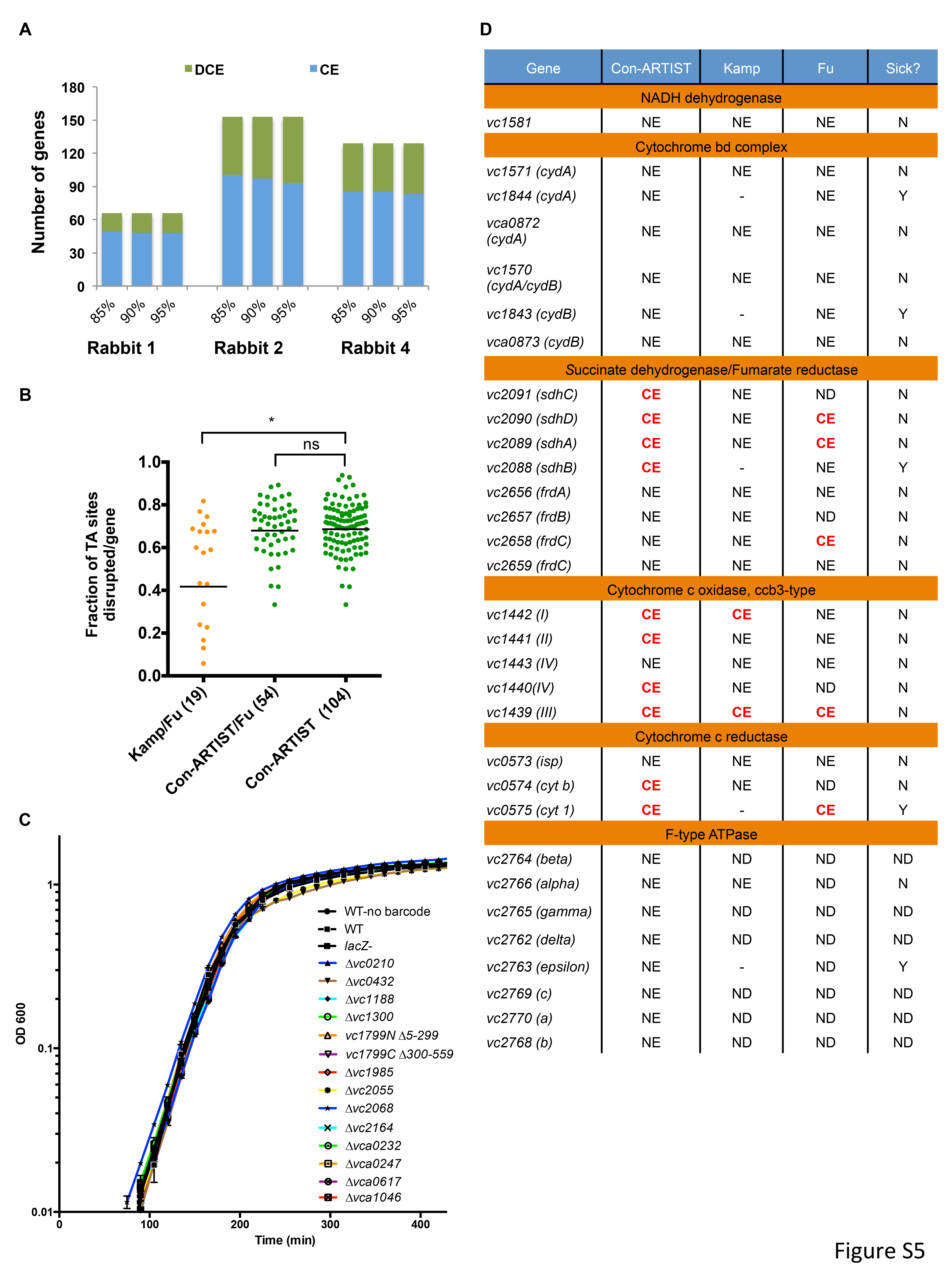

Supplement: Figure S5 — Con-ARTIST identifies conditionally essential genes in important pathways in V. cholerae. (A) In vitro and rabbit-passaged V. cholerae TIS data was analyzed by Con-ARTIST for each individual rabbit. Insertions were defined as conditionally essential if their probability of being assigned to this category exceeded the stringency cutoffs tested—85%, 90% or 95%. Insertions in each gene were combined and genes were then defined as conditionally essential (CE), in which every insertion probability has exceeded the stringency cutoff or domain conditionally essential (DCE), in which there are insertions in the gene that both exceed and do not pass the desired cutoff. In all animals, the total number of genes with conditionally essential regions stays the same regardless of the probability cutoff, though at higher levels of stringency some genes switch from being designated entirely conditionally essential to domain conditionally essential. (B) There were 19 genes that were similarly defined as conditionally essential by Kamp et al. and Fu et al., but were not found by Con-ARTIST (Figure 4C). These genes had significantly fewer disrupted TA sites in the in vitro input library compared to the 104 Con-ARTIST defined conditionally essential genes (*, p-value<0.005). (C) 13 candidate conditionally essential genes were deleted in V. cholerae, barcoded with a unique tag at a neutral locus and grown in LB alongside a tagged WT strain. Growth was monitored with OD600 measurements at 15-minute intervals. None of the deletion strains had an appreciable growth defect in vitro compared a non-barcoded WT strain that was grown in parallel. (D) V. cholerae conditionally essential genes found by Con-ARTIST, Kamp et al. [16], and Fu et al. [11] were mapped onto the predicted KEGG respiration pathway. Though all three studies defined genes in this pathway as being required for growth in vivo, Con-ARTIST found significantly more members than previous studies (p-value<0.05 by Fisher's exact te [file pgen.1004782.s006.tif]

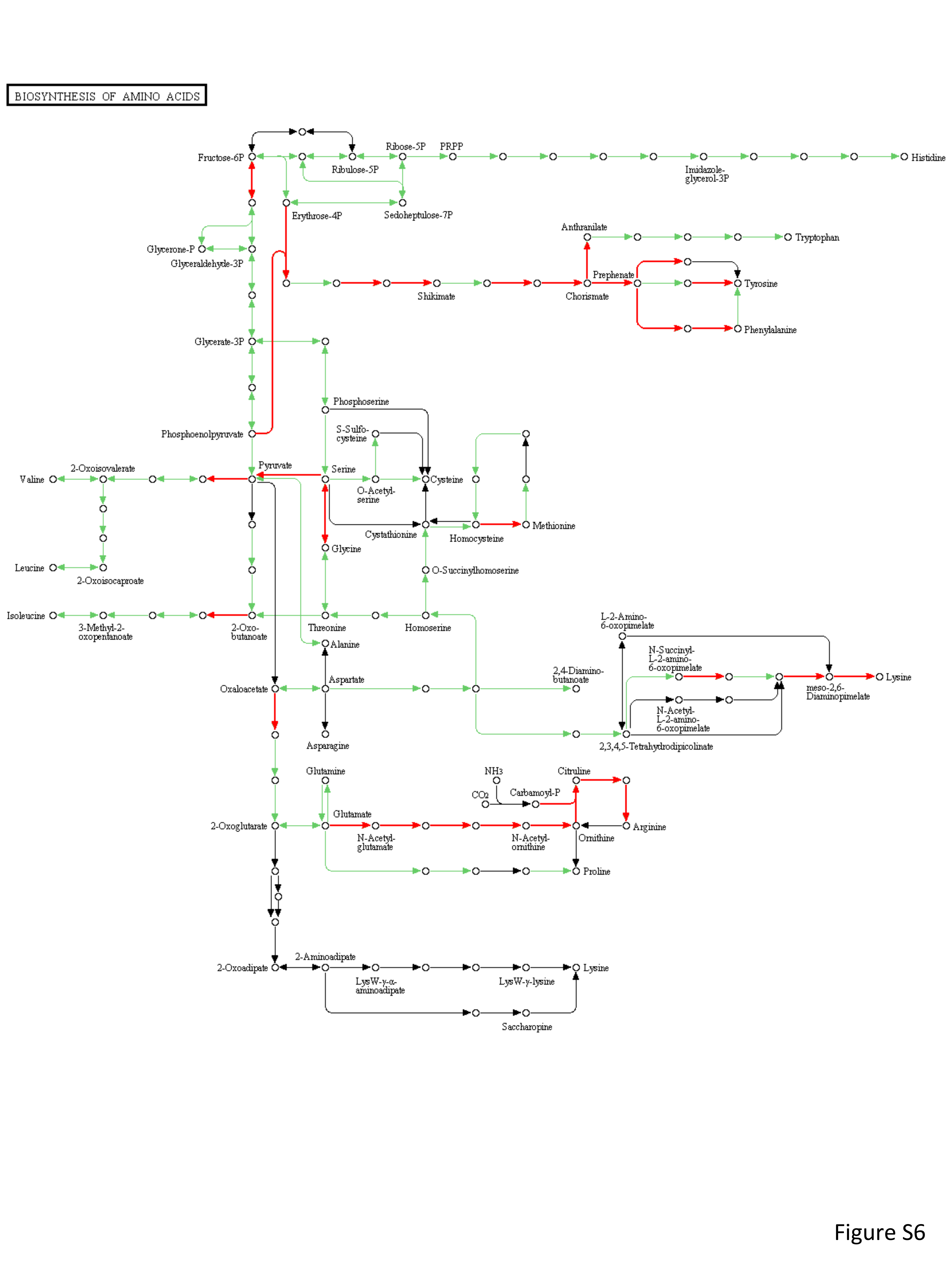

Supplement: Figure S6 — TIS studies highlight amino acid biosynthesis pathways required for V. cholerae growth in rabbits. Conditionally essential genes required for V. cholerae rabbit infection (but not for growth in rich media in vitro) that were identified by either Con-ARTIST, Kamp et al. [16] or Fu et al. [11] were mapped to the KEGG amino acid biosynthesis network map. Steps in amino acid biosynthesis catalyzed by genes that have homologues in V. cholerae are shown in green. Red arrows represent processes catalyzed by V. cholerae enzymes for which transposon mutants are underrepresented in vivo. (TIF) [file pgen.1004782.s007.tif]
