# Supplementary material for: ARTIST: High-Resolution Genome-Wide Assessment of Fitness Using Transposon-Insertion Sequencing
Source: PLoS Genet. 2014 Nov 6;10(11):e1004782. doi: 10.1371/journal.pgen.1004782 (PMC4222735; doi:10.1371/journal.pgen.1004782)
Supplement: Text S1 — The ARTIST user manual. The user manual provides background knowledge on the ARTIST pipeline and detailed instructions to carry out transposon-insertion sequencing analysis using the ARTIST scripts. (DOCX) [file pgen.1004782.s014.docx]

ARTIST: Analysis of high-Resolution Transposon-Insertion Sequences Technique

User Manual

**Table of Contents**

**I. Software and Data Requirements for running ARTIST**....................................3

**II. Schematic of the ARTIST workflow**...................................................................3

**III. General notes for use**........................................................................................4

**1. Creating a genome map of all mariner-insertion sites (TA dinucleotides)**...7

**2. Count the mapped reads at every TA site in the chromosome**................... 11

**3. Mapping Tn5 insertions into genome windows**............................................. 14

**4. Visualizing Sequencing Saturation and Bottleneck Effects**......................... 17

**5. Associate each insertion site to genomic loci**............................................... 20

**6. EL-ARTIST: Essential Loci Analysis**.............................................................. 21

**7. Con-ARTIST: Conditional essentiality analysis**............................................ 27

Appendices:

**A. Constructing a compatible GTF file in Excel from other formats.**.........31

**B. Using other programs to create ARTIST compatible data**......................34

**C. Running Con-ARTIST with resampling and MWU but without HMM**......36

**D. Output ARTIST results in other data formats**...........................................39

**References**............................................................................................................. 40

# Software and Data Requirements for running ARTIST

1. Matlab 2012b (or later) with the bioinformatics and statistics toolboxes. We recommend at least 4GB of available RAM on the computer. In the current paper, analysis has been run primarily on a computer with 16GB RAM. This pipeline has not been optimized for minimizing memory footprint.
2. Custom Matlab scripts from this publication (Supplementary Materials).
3. Pre-aligned reads from a Mariner-based or Tn5 –based transposon library in a SAM default output file format. There are separate workflows for using mariner and Tn5-based data (see below).
4. FASTA sequence files for each of the chromosomes that you have mapped your reads to.
5. Genome annotation file (in GTF or GFF2 format), which contains the name, start and ends sites for each annotated gene in the genome.

# Schematic of the ARTIST workflow


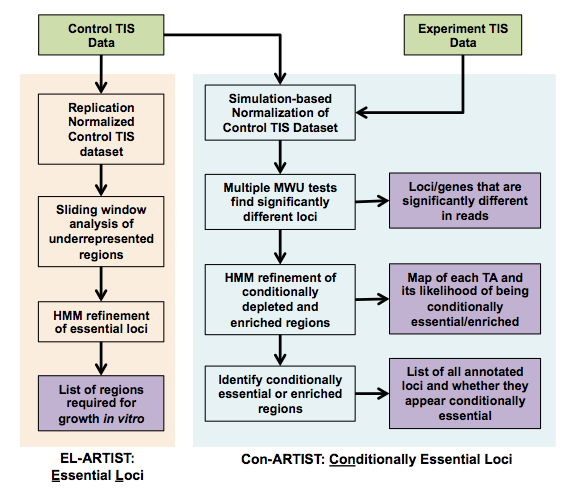


# General notes for use

1. Library complexity and sequencing

As both the EL-ARTIST and Con-ARTIST arms rely on confidently knowing the mutant composition of the control library, control libraries should be sequenced at a depth such that additional sequencing will produce minimal increases in the number of unique insertions mutants detected. See Section 4 to visualize how increases in sequencing depth might translate into the discovery of new transposon insertion events. In contrast, experimental libraries should be sequenced well, but sub-saturating depth can be tolerated, as simulation-based normalization compensates for differences between experimental and control libraries.

Both EL-ARTIST and Con-ARTIST pipelines utilize hidden Markov models (HMM), which work best with highly complex transposon libraries (i.e., when most potential insertions sites in have been disrupted). For EL-ARTIST, this requires construction of the most complex transposon library as possible to accurately predict the regions of essentiality. However, for Con-ARTIST, the library complexity you need will be determined by the severity of bottleneck in the experiment. This should be first empirically tested in your experimental system to guide library construction. We have included a script in Section 4 to model the effect of bottlenecks on the loss of unique transposon insertions and their impact on false positive discovery rates. For mild experimental bottlenecks, high-density libraries are preferred. In contrast, highly complex libraries will not provide analytical benefit if most insertion mutants are lost by chance at a tight bottleneck. In the latter scenario, it may be beneficial to use libraries of lower complexity in scale with the size of the bottleneck. Also, instead of using an HMM, it is preferential to analyze your data using an aggregative statistical method such as Mann-Whitney U analysis after simulation-based normalization for analysis (see Appendix 2).

Finally, it is difficult to define ideal and universal metrics for the number of reads and library diversity required for any given TIS experiment, as the effect of these parameters can be library and organism specific. However, we can report specifically from our studies in *V. cholerae* and *M. tuberculosis* that we were able to generate a relatively accurate TIS analysis using ARTIST when the starting TIS library contained insertions at > 60% of potential insertion sites and at least > 50% of these insertions were retained after selection in the host. Unique mutant discovery in control and experimental libraries began to plateau around 500,000 reads, and sequencing depths of 2-4 million reads typically produced 10-100 reads per neutral insertion, which was sufficient for Con-ARTIST analysis.

2. Analysis of biological replicates in ARTIST

For *in vitro* created libraries under optimal growth conditions, we have observed that the correlation of reads between independently created libraries is very high (R > 0.9), which suggests that making a single high-density transposon library is reasonable to reduce analysis time and increase the number of mutants that you can analyze at once.

However, experimental libraries selected on more stringent and/or uncharacterized test conditions (e.g., *in vivo* growth) can experience significant variability in terms of genetic drift. Thus, it is not recommended to pool these biological experimental replicates together, as each sample may be experiencing different jackpot events and bottlenecks. These conflicting processes can interfere with accurate downstream statistical analysis when combined. We found that for precise identification of conditionally essential loci, comparison of the control library to individual, rather than pooled, datasets yields more robust results. Consequently, all analyses presented were based upon comparison of libraries derived from individual animals versus the control, and reflect the consensus result between all animals.

3. Analysis of multiple DNA molecules

The ARTIST pipeline analyzes insertions across a single DNA molecule. It is possible to concatenate different chromosomes and plasmids together into a single template for analysis in ARTIST, but we have previously noticed that read counts from insertions on chromosome II in *V. cholerae* were consistently lower than read counts from chromosome I (1). Since each chromosome appeared to have a different relationship between the numbers of reads from each insertion and the fitness of each mutant, we analyzed each chromosome independently. We suggest re-running the entire ARTIST pipeline separately for each DNA template that you are analyzing, but changing the names of the output files so that they are not overwritten between runs.

4. Matlab outputs and inputs

ARTIST is created to work in the Matlab environment, which utilizes its own syntax and file structure (information can be found at <http://www.mathworks.com/products/matlab/>).

In this user manual, we have color coded the commands to run the scripts as follows:

Example:

[Output_1, Output_2] = function (Input_1, ‘Input_2’);

The commands you will type are shown in red and in the Courier font. The outputs of the script are located between square brackets to the left of the equal sign, while the function name is to the right. The scripts have a semi-colon at the end to suppress reporting of intermediate calculations and reduce the computation time of each script.

The yellow highlighted names between parentheses are input data that you will be required to provide for the script to run. In general, anything that is character-based will be placed between single quotes. The exception is variables/files that you have imported already into the workspace, which will be called by typing in the variable names without quotes.

Output files of the scripts are marked in blue and will appear as matrices that can easily be cut and pasted into Excel. You can also use Matlab output functions (Appendix D) to export these tables as text files.

We have included example annotation (‘VCgtf’) and Bowtie mapping files (‘VC.sam’), as well as a Matlab workspace (User_manual_examples.mat) to provide examples of real TIS data that have been processed by the ARTIST pipeline.

# 1. Creating a genome map of all mariner-insertion sites (TA dinucleotides)

This section will cover importing genome annotation GTF/GFF2 files into Matlab for downstream parsing and annotation of all the potential mariner-based transposon insertion positions in the genome. Note: for Tn5-based data, follow steps 1-7 below and then proceed to Section 3.

1. Open Matlab.


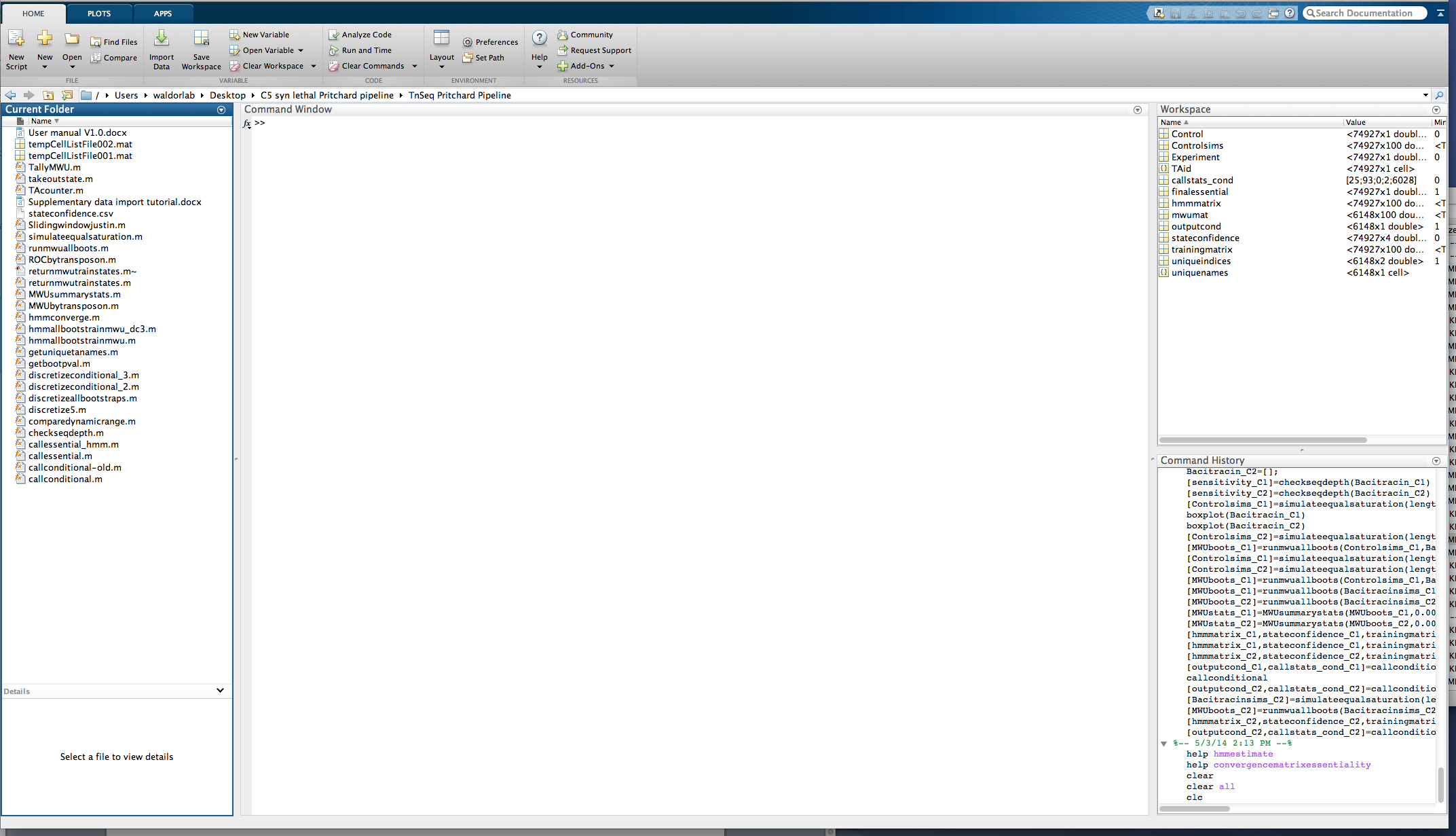


Command Window

Above is the default Matlab workspace: the current folder (or Matlab ‘path’) and associated files are on the left; the command window (center) is where you will type and initiate functions; the workspace (top right) contains the current variables (data) that you have imported or generated in the analysis.

Workspace

Current

Folder

1. To begin using ARTIST’s custom Matlab analysis scripts, you will need to make sure that the scripts are in the Matlab “path”. To change the path, simply click the address bar (arrow) and navigate to the folder in which the ARTIST scripts (and your bioinformatics files) are located on your computer. These scripts will show now show up in the ‘current folder’ window.


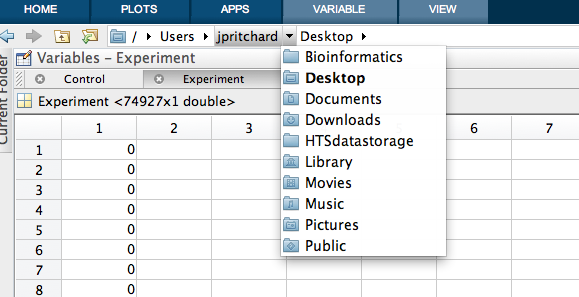


1. Download a GTF/GFF list of features for your organism. We have adapted our scripts for the GTF format used by the UCSC microbial browser (<http://microbes.ucsc.edu>). Note: there are additional repositories of genome annotation files, such as Ensembl Bacteria (<http://bacteria.ensembl.org/index.html>). However, other formats from different sources may have slightly different wording, which will prevent accurate data parsing by our scripts. Please see Appendix A for instructions in how to use Excel to change these other formats into a compatible GTF-like file that can be used in this pipeline.

There are at least 9 tab-delimited columns in a GTF file. The important columns in this example for downstream analysis are as follows:

1) Chromosome identifier;

3) Type of genomic feature (coding, start codon, exons, etc);

4) Leftmost coordinate of features (regardless of which strand/direction feature is encoded on);

5) Rightmost coordinate of features (our effective ‘end’ of feature);

9) Name of genomic features

1. Open the GTF file in Excel or a text editor and write down the name of the chromosome(s) of your organism in the GTF file. In this case (below), chromosome I is called ‘chrI’ (circle).


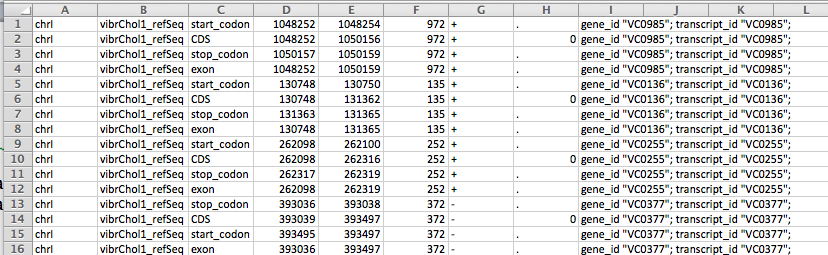


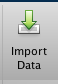


1. Click the ‘Import Data’ button, navigate to your GTF file and click open.
2. In the new import window, change the format (red circle) of the data to ‘Table’ (could be called ‘Dataset’ in older versions of Matlab’).
3. Change the headers and data type (arrows) of the relevant columns to 1) ‘Chrom’; 3) ‘Type’; 4) ‘Start; 5) ‘End’; 9) ‘Name’. Also, ‘Chrom’, ‘Type’ and ‘Name’ columns should be imported as ‘TEXT’, not ‘NUMBER’. Conversely, ‘Start’ and ‘End’ should be in NUMBER format. This is critical since the downstream function searches for columns named under these terms. **Note**: Matlab is case sensitive.


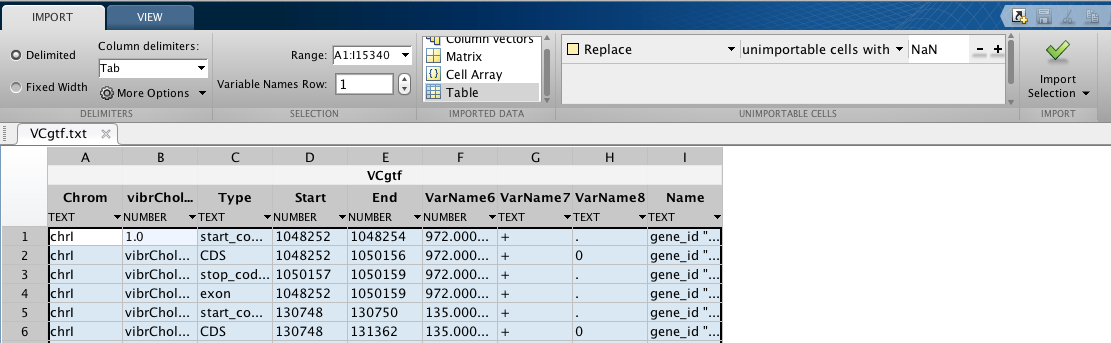


1. Hit ‘Import Selection’ (blue circle). The GTF file will now be put into your workspace labeled as the name of the file (e.g., VCgtf). **Note**: you may replace the name of the GTF file with anything you like, but you will have to substitute the new name in the appropriate places in all downstream commands.
2. Download a FASTA sequence file of your chromosome(s) and move them into the Matlab path (i.e., the folder in which the ARTIST scripts are located). They are in the path when the files appear in the ‘Current Folder’ window. Record the last nucleotide position of each chromosome (the length of chromosome).
3. Run the ‘genome_parser_TA’ script. Depending on the size of the GTF file and genome size and available memory, this script could take from 5-30 minutes.

Overall, this script will take the GTF table and look for all features called ‘CDS’ (e.g., protein coding genes). Then using a chromosome identifier (chr_search_term), it narrows the list to genes from a specific chromosome. The start and end positions for these genes are used to create the intergenic regions in the chromosome (there will be an intergenic (IG) locus if there is any space between the start of a gene and the end of the previous one). Finally, using an in-built ‘fastaread’ function in Matlab, the script will then search for all TA sites (or other motifs) in the sequence, mark the chromosomal positions at which they occur and then locate which genes or intergenic region they fall into.

Type:

[Chr1_names, Chr1_starts, Chr1_ends, TAsites, TAid] = genome_parser_TA (GTFtable, ‘chr_search_term’, chrom_end, ‘fasta’, ‘motif’);

For the function to run, you will need to provide several inputs (highlighted):

The ‘GTFtable’ input is the name of the GTF table that was imported into the Matlab workspace. Type in the table’s name without quotation marks.

‘chr_search_term’ is the name of the chromosome (as given by the program/site that made the original GTF file) that you are looking for. You found this in step 3 (e.g., ‘chrI’). You must type in the name of the chromosome exactly as it in the GTF table, surrounded by single quotation marks.

‘chrom_end’ is the last nucleotide position of the chromosome, no quotes.

‘fasta’ is the name of the FASTA sequence file of the chromosome you are working with. You will type in the name of the file, including whatever extension the file has (e.g., ‘sequence.fa’ or ‘sequence.fasta’), between single quotes. **Note**: make sure the FASTA sequence is in the Matlab path, or you will have to type in the full directory to locate the file (e.g., ‘User/ Desktop/ Folder…’ ).

‘motif’ is the sequence motif you are searching for, surrounded by single quotes. For mariner libraries, this will be ‘TA’.

Example command:

**[Chr1_names, Chr1_starts, Chr1_end, TAsites, TAid] = genome_parser_TA (VCgtf, ‘chrI’, 2961149, ‘VC_chr1.fa’, ‘TA’);**

The output of the script will consist of 5 arrays (Excel-like tables) that will show up in the Workspace after the script has finished. Double clicking on each array in the workspace will open them in the Command window.

‘Chr1_names’ will be list of all the gene and intergenic regions that exist on the chromosome. The list is in the order as you would encounter then on the chromosome moving from the first to last nucleotide.

‘Chr1_starts’ is a list that tells you the base position where each gene and interenic region from ‘Chr1_names’ starts.

‘Chr1_ends’ is a list with the end base positions of all annotated loci.

‘TAsites’ is the base position of all TA sites in the chromosome.

‘TAid’ is a list of which loci every TA site occurs in the chromosome. Each cell in this list corresponds to the same cell in the TAsites list.


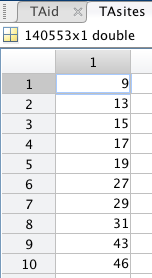

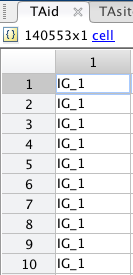


1.
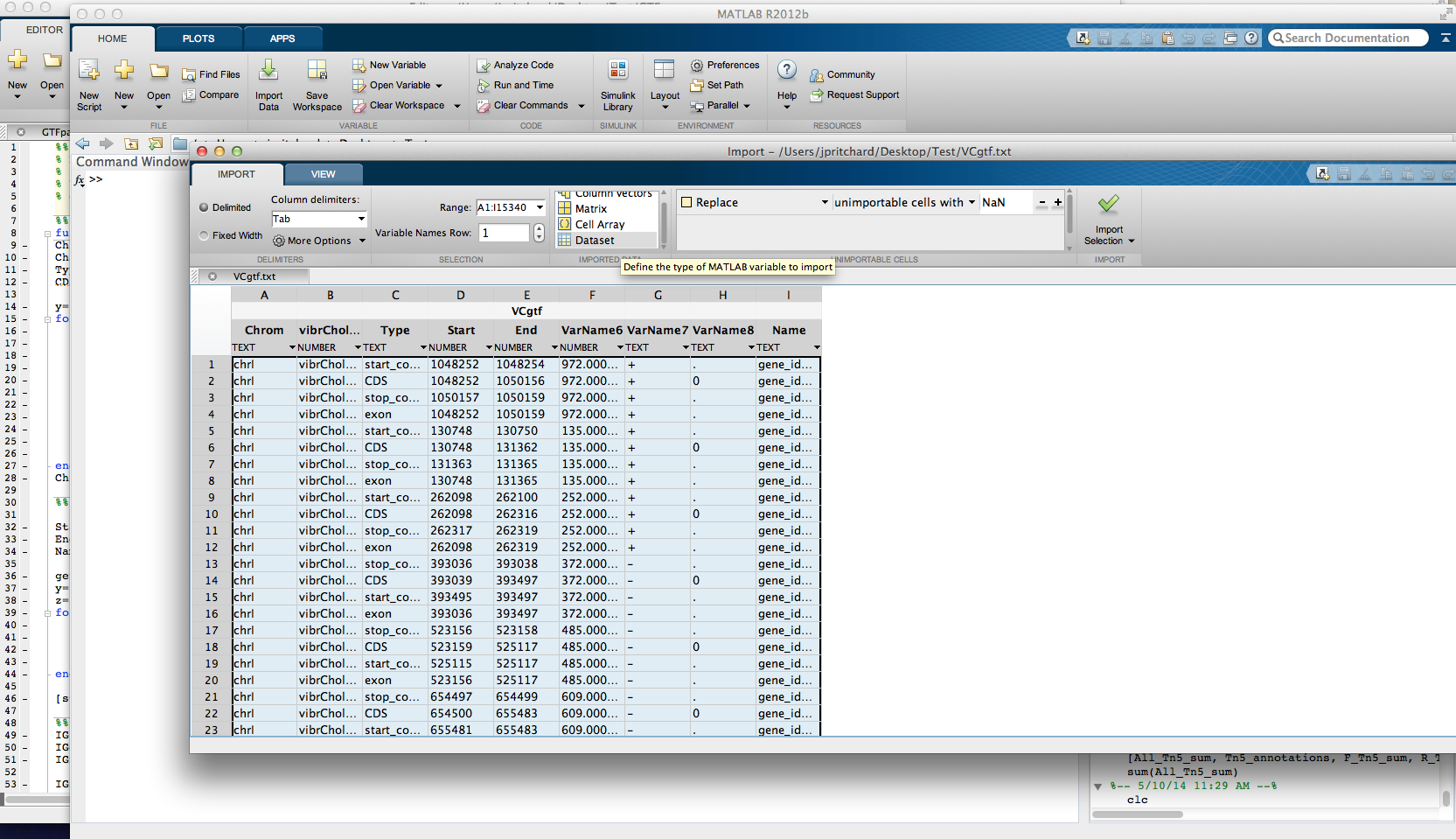
Save this workspace as a .mat file so you can re-use the data for all future TIS analysis in the same organism, as the TA positions and gene locations do not change between studies. **Note**: use the ‘Save workspace’ button to save all data, as simple save (e.g., ctrl-S) will only save the matrix that you are currently seeing in the command window.

Next time, you can import the saved .mat file into Matlab and skip this section altogether. With the data in the workspace, proceed to the next section.

# 2. Count the mapped reads at every TA site in the chromosome

This next function will analyze a SAM mapped reads file to determine where each insertion occurred and how many reads for those insertions were detected across the chromosome. **Note**: since we are only evaluating reads at TA dinucleotides, the SAM mapping positions must be accurate. Before mapping in Bowtie, reads should be trimmed for transposon-associated sequence but the duplicated ‘TA’ after transposition needs to be kept to make the numbers align. You will see that reads mapping to the plus strand will always begin with TA (arrows below).

1. Before using ARTIST, you will have mapped the trimmed sequenced reads to your genome (all chromosomes/plasmids) using Bowtie, and exported the data in SAM format (see instructions for Bowtie at <http://bowtie-bio.sourceforge.net/index.shtml>). The SAM format is a tab-delimited text file in which you can visualize your reads, where they mapped to, and sequence, etc. Some sequencing services may map your sequences for you and provide data in a compressed BAM format. This can be converted to SAM format using SAMtools scripts (<http://samtools.sourceforge.net>).

**Note**: the following function is specific for single end reads only. Do not use paired-end mapped SAM files as the reads will not be recognized by the script. If you have paired-end data, use only the Forward (first) sequencing FASTQ file for Bowtie mapping.

1. Move the SAM file into the Matlab path/folder.
2. You will need to copy down the name of the chromosome (red circle) that you mapped your reads to in Bowtie. This can be found in the SAM file when you open it with a text editor. Also, note that there are 3 FLAG codes to pay attention to (blue circles). 0 = the read was mapped on the plus strand (forward orientation); 4 = read was not mapped to the genome; 16 = read was mapped on the minus strand (reverse orientation). Our script only looks for reads with FLAG codes 0 and 16.


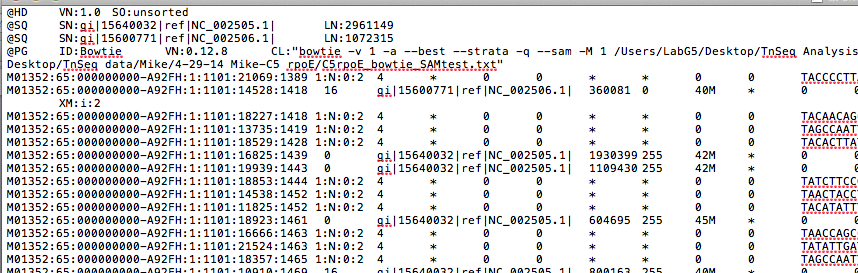


1. Run the ‘SAMreader_TA’ function.

**Note:** this script may take a long time, depending on the computer memory available and length of SAM file (number of reads that were used to map). On a 8GB machine, for ~5 millions reads, this script ran in ~2 hours, but you may have to leave the script running overnight. Because SAM files are large, we are reading in data in blocks of reads. We have found that 100,000 reads seemed reasonable for balancing computer memory availability and time of processing. However, depending on the machine, the number of reads that can be processed can be increased or decreased to your preference. The script will automatically calculate how many iterations it needs to run to get through the whole SAM file once you input the number of reads you want per block.

Overall, this function will count how many reads were sequenced from insertions at each of the TA sites in the chromosome. The output is now compatible with the ARTIST pipeline for analysis.

Type:

[Freads, Rreads, Total_reads] = SAMreader_TA (SAM_file, number_of_reads_blocked, chr_identifier, TAsites)

You will need several input variables:

‘SAM_file’ is the SAM mapping file you generated. Type in the name of the file, including its extension (e.g., ‘.sam’, ‘.txt’), surrounded by single quotes.

‘number_of_reads_blocked’ = the number of reads you want Matlab to process at a time. No quotes necessary.

‘chr_identifier’ is the exact name of the chromosome file you mapped your sequences to (red circle above). Type in the identifier between single quotes, including all punctuation and symbols. In the example above, ‘chr_identifier’ will be called ‘gi|15640032|ref|NC_002505.1|’.

‘TAsites’ is the matrix you created in the last function. Just type in the name of the variable (TAsites) without quotes.

Example command:

**[Freads, Rreads, Total_reads] = SAMreader_TA (VC_WT.sam, 100000, ‘gi|15640032|NC_002505.1’, TAsites);**

The function will provide 3 outputs:

‘Freads’ is a list for every TAsite in the chromosome and the forward reads detected at that site (i.e., reads from transposons that inserted on the plus strand).

‘Rreads’ is the same as Freads, but representing reads from insertions on the minus strand.

‘Total_Reads’ is the combined Fread and Rreads per TAsite in the chromosome. You will rename this as either ‘Control’ or ‘Experiment’ for downstream analysis, depending on which data was used for mapping.

1. Give your data a quick check to see if the tallying was accurate. The number of Freads and Rreads across a chromosome should be similar, since transposition is largely random.

Type:

Fread_sum=sum(Freads};

Rread_sum=sum(Rreads);

Total_reads_sum=sum(Total_reads);

Check the numbers in each of these new variables to make sure the numbers look as you would expect (e.g., Fread and Rread are about the same and add up to Total_reads_sum).

1. At this point, you have an array of every TA site in the chromosome (TAsites), the forward (Freads), reverse (Rreads) and combined (Total_reads) reads at each of these sites, and which locus in which those TAsites exist (TAid). This is the TIS data you need for ARTIST analysis.

Assign the data to your specific sample by typing:

Control=Total_reads;

or

Experiment=Total_reads;

# 3. Mapping Tn5 insertions into genome windows

ARTIST’s hidden Markov modeling (HMM) based functions rely on having a high apparent transposon saturation rate across the genome. In the case of mariner-based transposons, TA dinucleotide specificity means that there are ~100,000-300,000 potential insertion sites in the genome for most bacterial species. It is readily achievable to create libraries with insertions at most of these sites.

However, Tn5 does not have a specific site of insertion. The benefit of this is that the entire genome sequence is open for transposition. The drawback for HMM analyses is that even with high genome saturation (e.g., insertion every 10 bp), saturation will still appear low (10%) and since ARTIST’s statistical power relies on its ability to analyze TIS datasets with high saturation rates relative to the total number of potential transposon insertion sites, with most potential sites having no reads using Tn5, HMM analysis will have a hard time deriving a consistent read count signature across the genome.

To combat this issue and offer HMM accessibility to Tn5 data, we allow users to combine insertions into small genomic windows, where most windows will have reads. This boosts the apparent saturation of Tn5 data such that ARTIST can establish accurate signatures for HMM analysis.

1. You will first define the coding and intergenic sequences/positions from a GTF file as you would with mariner-based data (see Section A). Unlike Section B, you will run a different script, ‘genome_parser’.

Type:

[Chr1_names, Chr1_starts, Chr1_ends] = genome_parser (GTFtable, ‘chr_search_term’, chrom_end);

This script has several inputs that are necessary (highlighted), and produces several output files (between square brackets). These files are the same as those described in Section A.

Example command:

**[Chr1_names, Chr1_starts, Chr1_ends] = genome_parser (GTFtable, ‘chr_search_term’, chrom_end);**

1. Next, you will tally the reads from all insertions and assign them to small genomic windows using the ‘SAMreader_Tn5’ script. Note: SAM files are large, containing many sequences to be processed. Consequently, we are uploading SAM mapped reads in blocks and iterating through until all reads are processed. We have found that uploading 100,000 read blocks strikes a good balance of memory usage and time of processing. However, this script may hours to run depending on the computer’s available memory, the number of sequences mapped and the size of the genomic windows to be evaluated.

This script specifically creates non-overlapping windows across the genome according to a size that you choose. Then, using the SAM mapping file (and the ‘samreader’ function in Matlab), all the reads from insertions in each window will be tallied. The reads for each window will then be arbitrarily assigned to a genome position in the middle of that window (e.g., reads in a window covering nucleotides 1-100 will be assigned to a position of 50). Finally, the new window positions will then be used to assign the window to a locus (e.g., Gene A).

Type:

[Tn5_NT, All_Tn5_sum, Tn5_annotations, F_Tn5_sum, R_Tn5_sum] = SAMreader_Tn5 (Chr1_ends, Chr1_starts, Chr1_names, SAM_file, number_of_reads_blocked, window_size, chr_identifier, Genome_Pos);

The function requires several inputs:

‘SAM_file’, ‘number_of_reads_blocked’, and ‘chr_identifier’ are the same entries that would be used for the mariner-based SAMreader script (see Section A).

‘Chr1_ends’, ‘Chr1_starts’, and ‘Chr1_names’ are the outputs created from the genome_parser script. Type in the names of these arrays, without quotes.

‘window_size’ is the size of the region (in nucleotides) in which all insertions will be combined.

‘Genome_Pos’ is the last nucleotide (length) of the chromosome.

Example command:

**[Tn5_NT, All_Tn5_sum, Tn5_annotations, F_Tn5_sum, R_Tn5_sum] = SAMreader_Tn5 (Chr1_ends, Chr1_starts, Chr1_names, ‘VC_WT.sam’, 100000, 100, ‘gi|15640032|NC_002505.1’, 2961149);**

The outputs of the function:

‘Tn5_NT’ is a list of the midpoint positions of the windows created. This will serve as the equivalent of TAsites (see Section A), which we can also use for replication read correction.

‘Tn5_annotations’ is a list of loci in which each window midpoint is located. Each cell in Tn5_NT matches the same cell in Tn5_annotations.

‘F_Tn5_sum’ are the reads from all insertions within each window. Each cell in this column corresponds to the window position in the same cell in ‘Tn5_NT’.

‘R_Tn5_sum’ are the reads form all insertions within each genomic window.

‘All_Tn5_sum’ are the total reads for insertions within each window. This will be the data that you will use in the ARTIST pipeline.

1. Check your new data to make sure values are being assigned correctly. The Fread_sum and Rread_sum should be similar since insertion on each strand is random.

Also, double click on the ‘All_Tn5_sum’ array to visually evaluate whether the window size you chose is appropriate for your data. You want to find the cells that correspond to a non-essential gene (look in ‘Tn5_annotations’ for locus names) and see whether a majority of the windows have reads. If your window is too small (i.e., your library is undersaturated) you will see many 0’s in non-essential loci. Conversely, for highly saturated libraries, you can reduce the window size to maximize genomic resolution without creating large stretches absence of reads.

Type:

Fread_sum=sum(F_Tn5_sum);

Rread_sum=sum(R_Tn5_sum);

Total_reads_sum=sum(All_Tn5_sum_reads);

1. At this point, you will have list of all windows (represented by their mid-point position) associated with a specific locus. The All_Tn5_sum matrix represents all the reads from insertions that occur in each genomic window. Depending on your input file, this will be either your Control or Experimental data to use in ARTIST analysis.

Assign the data to your specific sample by typing:

Control=Total_reads;

or

Experiment=Total_reads;

# 4. Visualizing Sequencing Saturation and Bottleneck Effects

A. Sequencing Saturation:

ARTIST analysis follows the assumption that you have deeply sequenced your control library. We assume that despite adding more reads, you will not discover many more unique insertions. Thus we can make the assumption that given a sequencing depth, we know the “true” frequency of every insertion mutant in the library.

The ‘checkseqdepth’ script simulates taking N read counts randomly from your library and assesses how many unique insertions were detected. N increases from 1 to the total number of reads in your library in increments of 100,000. The number of unique insertions (e.g., number of TA sites, or genomic windows disrupted) is then plotted on the Y axis against reads sampled on the X axis. Each point is an average of 10 independent simulations.

Type:

[sensitivityanalysis] = checkseqdepth(Control);

The graph below will show up. If you do not save this graph, running the script again on another dataset will overwrite the current graph. Also, you can copy and paste the data from the new ‘sensitivityanalysis’ matrix and replot in Excel for ease (sampled reads [X axis] are in column 1; # of unique insertions found [Y axis] are in column 2).


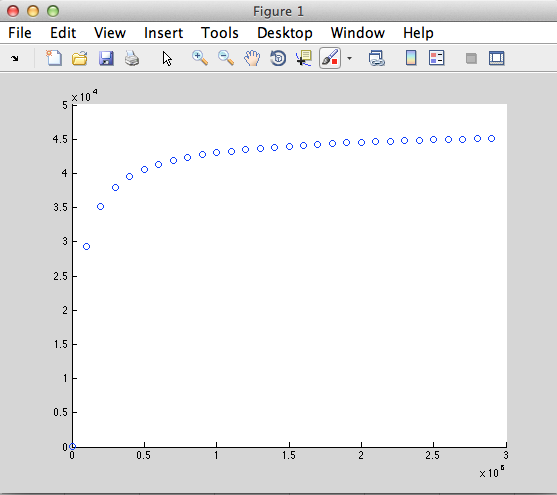


# Unique Insertions Found

# of Reads Sampled

Note the large degree of saturation in this dataset as the number of reads approaches 3 million. This indicates we are unlikely to find many more unique insertion mutants if we sequence deeper. Thus, this dataset can be used as a valid Control for TIS analysis.

B. Bottleneck Simulations:

ARTIST’s multinomial based normalization of TIS data is designed to reduce the impact of noise from bottleneck events on downstream analyses methods. However, it is critical to understand whether the bottleneck in our experimental condition is so severe that we might expect the noise to contribute to many false loci assignments in spite of normalization. We can estimate this impact using the ‘Bottleneck_analysis’ script included in ARTIST.

This script utilizes the Control (input) dataset and passes the library of reads through bottlenecks of certain sizes provided by the user (typically, the proportion of mutants that were actually lost in the Experimental dataset). After passage through a bottleneck, a proportion of unique insertions will be removed from the Control library by chance. The reads from the remaining mutants are tested against the original library (after being adjusted to the same total reads using a multinomial distribution or multiplying reads with a single scaling factor) with Mann-Whitney U statistical tests. This analysis passages the mutants through a bottleneck 10 independent times and generates 10 separate results in the same matrix.

Since bottleneck-passaged libraries originate from the Control data, no genes should be significantly different in reads in these tests. However, as bottleneck severity increases, this false positive rate will rise, and it is important to assess whether the false positive rate we observe in our simulations is dramatically higher than we would like to tolerate. If the bottleneck is too severe, we expect to identify many false positive genes due solely to the genetic drift inherent in the study. Utilizing a lower diversity library or an experimental system with a wider bottleneck would be preferred in this instance for more robust TIS results.

To run the script, type:

[bottleneck]=Bottleneck_analysis(Control, bottle, reads, uniquenames, uniqueindices, pval, prop_sig);

The necessary inputs are as follows:

‘Control’ is a matrix containing your mapped reads to each insertion site.

‘bottle’ is the proportion of the TIS library that will be **retained** after the bottleneck (e.g., use 0.8 for 80%).

‘reads’ is the total number of reads you want to normalize to. Typically this number is the total number of reads in the control library.

‘uniquetanames’ and ‘uniqueindices’ are matrices generated in Step 5 (below) where each insertion site is associated with a specific genomic feature (e.g., gene or intergenic region).

‘pval’ is the numerical p-value threshold for considering a locus significantly different in reads (e.g., ‘0.01’).

‘prop_sig’ is the fraction of Mann-Whitney U tests (MWU) that have to be significant (below the pval threshold above) in order to be considered a false positive assignment. In the multinomial distribution, we simulate 100 different libraries for each bottleneck. Each of these libraries’ reads are tested against the original by MWU tests for statistical significance. We would use ‘0.9’ here to call genes as falsely significant if over 90% (> 90 of 100 tests) of the MWU tests performed had a p-value lower than the number we provided above.

Example command:

**[bottleneck]=Bottleneck_analysis(Control, 0.8, 1000000, uniquenames, uniqueindices, 0.001, 0.9);**

The output of the script:

‘bottleneck’ is a table that contains two columns, 10 rows each. Each row represents an independent bottleneck simulation. The first column is the number of false positive loci that were found after passing the starting library through a bottleneck and normalizing reads using a multinomial distribution (preferred). The second column is an estimation of the number of false positive genes identified if we had passed the library through the bottleneck, but normalized using a simple multiplicative scaling factor without a multinomial distribution. The latter values only serve to appreciate how robust multinomial-based normalization can reduce noise in the analysis. Averaging the values in each row will provide an average false positive discovery metric, while the standard deviations in the numbers represents the variance of passing a library through 10 independent bottlenecks of the same size.

Note: This script may take a long time to run (> 1 hour) depending on the number of datapoints (e.g., TA sites) and reads in the dataset. Also, the number of false positive assignments from this script is only an estimation of the severity of the impact of the experimental bottleneck, but it is not a true calculation of the false positive rate of Con-ARTIST, as it does not account for loss of truly attenuated mutants nor does it include downstream hidden Markov modeling.

# 5. Associate each insertion site to genomic loci

Each (potential) insertion site in the genome will now be linked with their associated annotated genomic feature. This allows ARTIST to keep track of the genomic position of all transposon insertions in the library.

1. If you used a mariner-based dataset, the ‘TAid’ array created by the ‘genome_parser_TA’ script will be used directly. Skip down to step 2.

If you have Tn5 data mapped to windows, just rename the ‘Tn5_annotations’ array to ‘TAid’ (below).

Type:

TAid=Tn_annotations;

1. Next, we will tell ARTIST where each insertion is in the chromosome, and to which loci they belong.

Type:

[uniquenames,uniqueindices]=getuniquetanames(TAid);

This creates two ordered lists of unique names and indices for your genome:


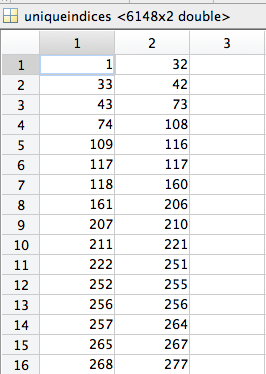

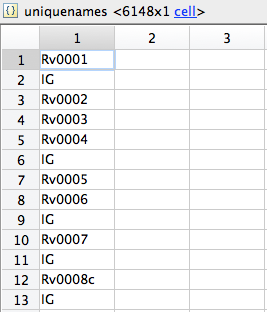


The ‘uniquenames’ matrix lists every unique locus in order from the start to end of the chromosome.

The ‘uniqueindices’ matrix shows which insertion sites belong to each locus in the chromosome. Every row is in the same loci in the same order as in ‘uniquenames’, and the numbers refer to the first and last insertions (row numbers) in the Control and Experiment arrays.

In the example above, the gene Rv0002 is comprised of 30 potential TA sites. The reads from these insertions are in rows 43-73 in the Control and Experiment matrices.

# 6. EL-ARTIST: Essential Loci Analysis

The EL-ARTIST pipeline identifies regions that are required for optimal growth under a given condition (typically an *in vitro* grown library on rich medium). The workflow begins by normalizing the data for incomplete DNA replication. Then, sliding window analysis is used to define regions that appear underrepresented in reads (2). Finally, the results of the sliding window analysis are used to train a hidden Markov model, which will refine the prediction of each TA site as being in a region required or dispensable for growth (1).

1. Essential Loci analysis begins by correcting for positional biases in the read count data. In fast growing bacteria, multiple origin firings and incomplete rounds of DNA replication create a gradient of reads from transposons near the origin towards the terminus (below). This bias must be normalized so that read counts truly reflect the fitness of transposon mutants across genome and not their DNA replication state.

First, check your data to see if you have an observable replication bias across the chromosome.

Type: scatter(TAsites,Control);

In example below, there is a symmetric ‘V’ shape in the raw data, indicating reads at origin proximal sites are more abundant than at the terminus. Thus, we will take 100kb windows across the genome, average the reads within each window and derive a local scaling factor compared to the average reads of the whole chromosome (3). Then reads in each window are multiplied by their scaling factor to normalize the local positional bias (3).


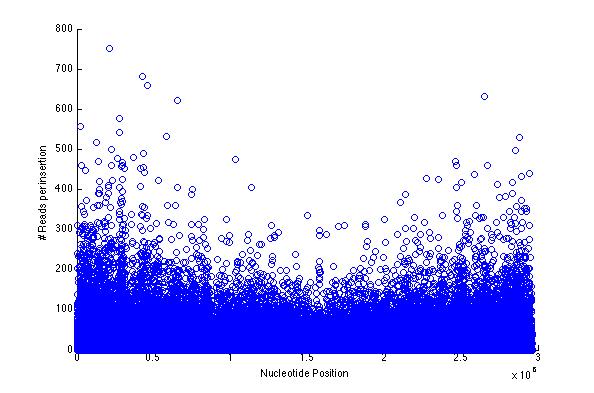

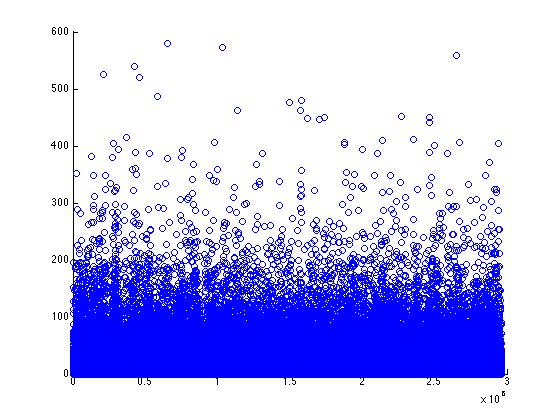


Chromosomal position (nt)

Reads per insertion

Reads per insertion

**Raw Data**

**Normalized Data**

Chromosomal position (nt)

Type:

[normalized_reads] = window_average(TAsites, Total_reads, windowsize, genome_length);

You will feed in 4 inputs:

‘TAsites’ is the TAsites matrix you have previously generated in Section A. For Tn5 data, this will be the ‘Tn5_NT’ array you generated in Section C, so type Tn5_NT instead of TAsites.

‘Total_reads’ is your re-named ‘Control’ array.

‘windowsize’ is how many nucleotides you want to average reads across for normalization. Since we would like to avoid bias due to large operons of essential genes, we typically use 100,000 nucleotides per window, but you can increase or decrease this as desired.

1. Next, we will find regions that are underrepresented in reads in the Control dataset using a sliding window approach. This method takes windows of consecutive insertion sites and determines whether the reads in each window are significantly underrepresented when compared to the distribution of reads in all windows across the genome.

Type:

[essentialregions,essentiallist,bootstats,essentialpvals] = Slidingwindow (Control, 1000, 7, uniquenames, uniqueindices, 0.03);

The script utilizes should take 2-3 minutes to run and requires the following inputs:

1000 is how many independent simulations the script will perform;

7 refers to the sliding window size (e.g., 7 consecutive TA sites or 7 Tn5 windows)

0.03 is the p-value threshold for calling a region significantly underrepresented in reads. In this example, 0.03 was the probability of getting 7 consecutive 0’s in our *M. tuberculosis* Control dataset. This number should be changed to suit other datasets (see below).

The following are the output matrices of the script:

Bootstats is a vector whose first row is the number of reads in the bootstrapped window, and the second row is the proportion of TA sites without reads.

Essentialregions is a list of every TA (insertion) site and whether that site was defined as being non-essential (0) or essential (1) by sliding window analysis.

Essentiallist is a list of every annotated feature (e.g., gene and intergenic elements) and whether that feature is non-essential (0) or essential (1) by sliding window analysis. Specifically, the feature is only called essential (1) if all windows in feature were called essential in all simulations.

Essentialpvals is a list of each TA site and its significance value in 1000 simulations for being underrepresented in reads.

Note: You may have to use different p-value parameters in this script, depending on the transposon saturation level and sequencing depth of your data. For example, if the window is too small, the insertion density in your library too low, or the p-value threshold you chose too stringent, it becomes likely that no region appears significantly underrepresented by sliding window analysis. You will know this has happened when your Min and Max values in the essentialregions matrix are both 0. Reduce the stringency of your cutoffs and repeat the analysis until regions are both called non-essential and essential.


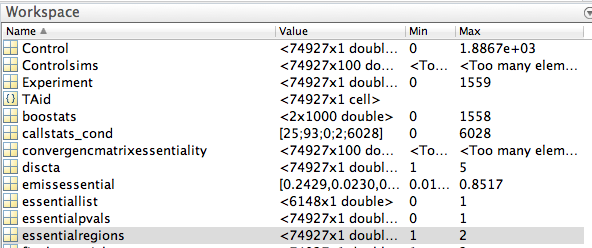


1. The sliding window essential regions found above are next used to train a hidden Markov model (HMM) to refine the essential regions. But first, you must add one to every essentialregions entry to make a training set for the HMM (the code will not recognize zeros).

Type: essentialregions=essentialregions+1;

Now, non-essential insertions = 1, while essential insertions = 2 (red box above).

1. Next, you will create appropriate cutoffs for discretization of the reads. Since PCR amplification during library preparation can mean that the exact number of reads detected for a mutant may not be completely accurate (e.g., an insertion with 90 reads may be in the same biological category as an insertion with 100 reads). Thus, we will discretize the reads of each insertion into more inclusive bins that are more likely to truly represent different biological categories.

We use a boxplot to judge how our reads will be grouped.

Type: boxplot(Control)


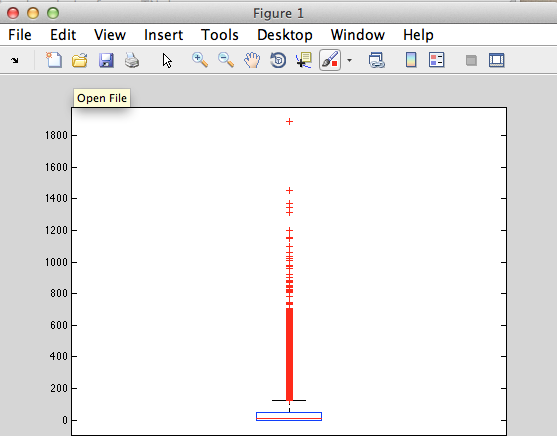


The red + signs denote insertions with reads that are beyond the boundaries of what the boxplot function considers outliers (see Matlab documentation on the boxplot function for more information).

The blue box represents the 25-75^th^ percentiles of reads, while the red line is the median reads per insertion. In most of the data we have analyzed, the boxplot appears compact: many values are zero (likely essential regions), and the median is usually 1 read or so. You may hit the zoom tool to find the values—they will be integers. As such, we will discretize reads according to this boxplot using the median, 25^th^ percentile, 75^th^ percentile and the outlier boundary values as cutoffs.

We will group our insertions into 5 bins: 1) group 1 will contain insertions with reads between 0 and the median (red line); 2) group 2 contains insertions with median reads (red line); 3) group 3 spans reads between the median (red line) and 75^th^ percentile (upper blue line); 4) group 4 covers insertions between the 75^th^ percentile (upper blue line) and the outlier boundary (top black whisker); and 5) group 5 covers insertion with reads greater than the outlier boundary (top whisker).

Type:

[discta] = discretize5(Control,0,1,7,17);

In the example above, the control has been split into 5 bins using 0, 1 (median), 7 (75^th^ percentile) and 17 (outlier boundary) as the cutoffs.

The output matrix ‘discta’ is a list of every insertion in the genome in which the reads have now been converted to a bin between 1—5 . In this example, insertions with 0 reads = 1; 1 read = 2; 2 to 7 reads =3; 8 to 17 reads = 4; and >17 reads = 5.

1. Next, use the discretized data and the sliding window essential regions to calculate the HMM’s transition and emission probabilities using the Baum-Welch algorithm.

Type:

[transessential, emissessential] = hmmestimate (discta, essentialregions);

1. Next, we use the transition probabilities generated above to train the HMM and predict the essentiality of every TA site in the genome.

Type:

[convergencematrixessentiality] = hmmconverge (discta, transessential, emissessential);

This script will run the HMM through all the TA sites (or windows for Tn5 data) in the genome, and based on the reads at these sites, predict whether the TA is located in an essential or non-essential region. After the first refinement, the HMM will recalculate the transition probabilities based on the new data and repeat the run. This continues until the algorithm reaches convergence, where rerunning the HMM on the data will produce the same result.

The output matrix, ‘convergencematrixessentiality’, contains the essentiality prediction for all TA sites in the genome (rows). Each column represents the predictions for each HMM run performed (below).


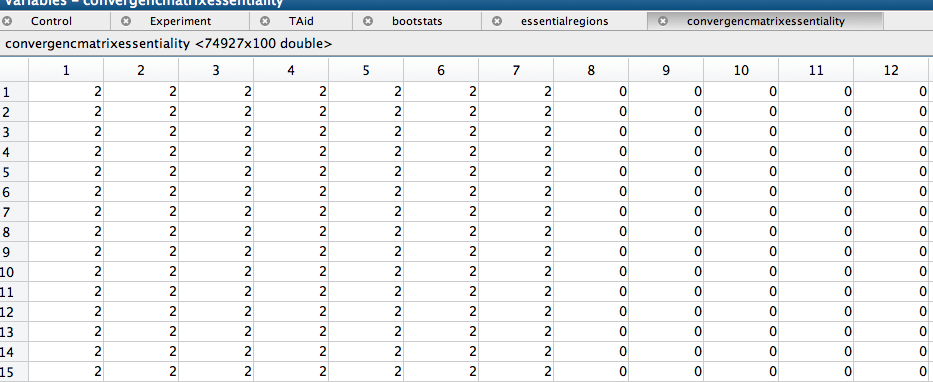


The final column (red box) in this matrix before the 0’s is the final HMM prediction for all TA sites in the genome. 1 = insertion sites that are in non-essential regions; 2 = sites in regions required for optimal growth.

9. Assign the final HMM prediction for all TA sites.

Type:

finalessential=convergencematrixessentiality(:,7);

This retrieves all of the rows (by using ‘ : ‘ ), in the 7^th^ column and places them in a new matrix named ‘finalessential’.

From this output, you can visually scan across each insertion to define regions that appear to be essential for optimal growth. Or go to the next step to combine all TA assignments into annotated loci.

10. Report the essentiality of all annotated loci.

You can simplify the analysis and output the results as a matrix for each annotated genomic feature (the loci in uniquenames).

Type:

[essential_calls, callstats] = output_essential (finalessential, uniquenames, uniqueindices);

The resultant table in ‘essential_calls’ is a matrix with a single column, where every cell refers to the locus in the same cell in the ‘uniquenames’ matrix. Each cell has a code, where 1 = the locus is non-essential for growth; 2 = essential for optimal growth; 3 = the locus is domain essential (contains both regions that are required and dispensable for growth).

The ‘callstats’ output is a simple count of all the loci that are called non-essential, essential and domain essential.

You can copy and paste the names from ‘uniquenames’ next to their HMM predictions from ‘essential_calls’ into a new spreadsheet to work with.

# 7. Con-ARTIST: Conditional essentiality analysis

This pipeline compares two TIS libraries grown under two different conditions and finds loci that are conditionally underrepresented or enriched. First, simulation-based resampling is used to normalize the Control data to account for random loss of mutants during the experiment. Then, reads in each locus are compared between datasets using a Mann-Whitney U test as was previously done (4). The MWU results are used to train the HMM, which will predict whether each TA site is in a region required for growth in a given condition.

1. Simulate genetic drift and sampling error in the experiment.

Since we are using as many sequences of the Control as we possibly can, we want to create the effect of equal saturation between the Control and the Experiment datasets. We do this by creating simulated controls that are normalized by TA density (as a measure of the severity of library bottlenecking/variability in the experiment) and sequencing depth (to normalize total reads quantitatively).

We simulate controls to approximate a bottleneck where a fraction of the TA sites are lost using a multinomial distribution. As an analogy, this creates a biased die with one side for each TA site, and the likelihood of landing on that side is proportional to its representation in the library.

Type:

[Controlsims] = simulateequalsaturation (length(Experiment(Experiment~=0))/length(Control(Control~=0)), sum(Experiment), Control, 100);

The length(Experiment(Experiment~=0))/length(Control(Control~=0)) input gives the relative proportion of TA sites disrupted in the experimental library versus the control.

The sum(Experiment) parameter gives the total number of reads to simulate.

100 is the number of simulations to run. You may run more if you like—we found that 1000 was no different than 100 in our data, and the drawback with more simulations is lengthier computation time downstream.

Note: Since we are comparing two datasets, both the Control and Experimental libraries should either be normalized for replication bias (Section D1), or both libraries should be the uncorrected raw data. We used raw data for our analyses here.

1. Define the read count discretization cutoffs that will be used to train the HMM.

Type: boxplot(Experiment)

A figure seen in pg. 24 will appear. You will choose the same cutoffs for the Experiment data: median, 75^th^ percentile, outlier boundary.

Note: We have found that some experimental libraries with low sequencing depth have a median of 0. In these cases, we arbitrarily chose ‘1’ as the median to allow the analyses to proceed.

1. Carry out MWU analysis for each locus and then use the results to train the HMM to predict conditionally essential/enriched regions. The following script may take a while to run, depending on the available processing power of the computer, and the number of insertions to analyze. Typically, it takes 15-30 minutes on a 16GB computer.

Type:

[hmmmatrix,stateconfidence,trainingmatrix] = hmmtrainmwu (Experiment, Controlsims, finalessential, uniquenames, uniqueindices, 0.01, 2, 5, 10, 1, 3, -1, -3);

In the script, 2, 5, 10 refer to read count discretization cutoffs (median, 75^th^ percentile, outlier boundary) derived in step 2.

The 0.01 parameter refers to the MWU p-value cutoff for defining genes with significantly different reads. Note: for genomes with fewer TA sites/insertions (e.g., high GC organisms like *M. tuberculosis*), 0.01 was appropriate. However, we found that more stringent cutoffs (0.001 down to 0.0005) produced more accurate training in organisms with many TA sites/insertions in the genome (e.g., *E. coli* and *V. cholera*e).

Finally, 1,3 and -1,-3 are the log2 fold change (corresponding to >2 fold and >8 fold) cutoffs for defining enrichment and depletion, respectively. The 1 and -1 refer to a required >2 fold change to define an enriched region, while 3 and -3 tells the HMM only call depleted loci if they are > 8 fold different in reads. You can use 2 and 4 (for 4 fold and 16 fold differences) in the HMM for added stringency.

There are 3 output files in this script:

The stateconfidence matrix represents every TA site in the genome (row). In the four columns are the probabilities of that TA site being put in the following categories across 100 MWU/HMM simulations: Column 1 is the likelihood the TA is not required for growth in either condition; column 2 = likelihood of being essential under both conditions; column 3 = probability of being enriched (overrepresented) in the experiment; column 4 = depleted in the experiment (i.e., conditionally essential for optimal growth).

You can visually scan across all insertions in the stateconfidence matrix to define loci that are conditionally required for growth. You can also aggregate calls across known loci (below).

The hmmmatrix output represents every TA site in the genome (row) and what the HMM believes is the most likely state for that insertion. The state is represented by number 1-4, which match up with the categories above. Each column represents the results of an independent Con-ARTIST run.

The trainingmatrix output represents every TA site in the genome and the number 1-4 signifies which biologic category (see above) that insertion was assigned to by the MWU test. Each column represents an independent MWU test.

1. Report the conditional essentiality/enrichment of all annotated loci.

After the HMM runs, we can use all probabilities for each TA to produce a final HMM assignment for the annotated loci in unqiuenames.

Type:

[outputcond,callstats_cond] = callconditional (stateconfidence, uniquenames, uniqueindices, 0.5, 0.9, 0.5, 0.9, 0.1);

In the following order, the input numbers refer to the ‘minsl’ (0.5), ‘maxsl’ (0.9), ‘minenr’ (0.5), ‘maxenr’ (0.9), and ‘propignore’ (0.1) values in the script.

Specifically, ‘minsl’ is the minimum probability cutoff for a TA site being called conditionally essential gene; ‘maxsl’ is the probability threshold for being called conditionally essential; ‘minenr’ is minimum cutoff for an enriched gene; and ‘maxnr’ is the threshold for calling an enriched gene.

‘propignore’ is the fraction of the 5’ and 3’ sequence of a locus to ignore in the analysis (e.g., to analyze the core 80% of a gene, you would put 0.1). This is used to remove potential interference of insertions within the extreme ends of genes, which may not disrupt gene function.

In the example script above, the probabilities of insertions in the core 80% of all loci will be considered. Loci in which all core insertions have conditionally essential (CE) probabilities >0.9 (in the stateconfidence matrix) will be called conditionally essential. Similarly, loci with insertions that have enriched probabilities >0.9 will be called enriched.

However, if a locus has insertions with CE probabilities >0.9 and also CE probabilities <0.5, then we believe this genes may have both non-essential and CE domains, so we will call this a domain conditionally essential locus (DCE). The same logic applied to domain enriched loci.

The output matrix outputconditional is a single column that contains the Con-ARTIST prediction (1-5) for the loci in the uniquenames matrix. The Con-ARTIST assignments are as follows: 1 = the locus is domain conditionally essential; 2 = the locus is entirely conditionally essential; 3 = domain conditionally enriched, 4 = entirely conditionally enriched; 5 = the region is not different between conditions (this may mean the region is either non-essential or essential in both conditions). You can compare the 5 calls to that from EL-ARTIST to distinguish between these two states. Each locus should be examined in the stateconfidence matrix to evaluate both the variance in probability across the locus and determine where in the locus transitions between states occur.

‘Callstats’ provides a count of how many loci in the chromosome fall into each biological category. Its order is the same as the label numbers in ‘outputconditional’ matrix, so please paste ‘unique names’ list next to this dataset in excel.

# Appendix

# A. Constructing a compatible GTF file in Excel from other formats

Not all genome annotation files will adhere to the UCSC GTF format that we used to create our scripts. However, you can use Excel to easily create a GTF-compatible Excel file that will work with our scripts.

1. Download and/or open a gene annotation file in Excel. The annotation file should have every gene, their names (e.g., ‘*rv0001*’), start positions, end positions, and the chromosome(s) on which they are located. The figure below is a GFF3 file for *M. tuberculosis* that was downloaded from Emsembl Bacteria (<http://bacteria.ensembl.org/index.html>). Note: though we use the terms ‘start’ and ‘end’, the positions actually refer to the leftmost and rightmost coordinate of the genes, regardless of whether it is actually encoded on the plus or minus strand (i.e., ‘start’ numbers are always smaller than ‘end’ numbers).


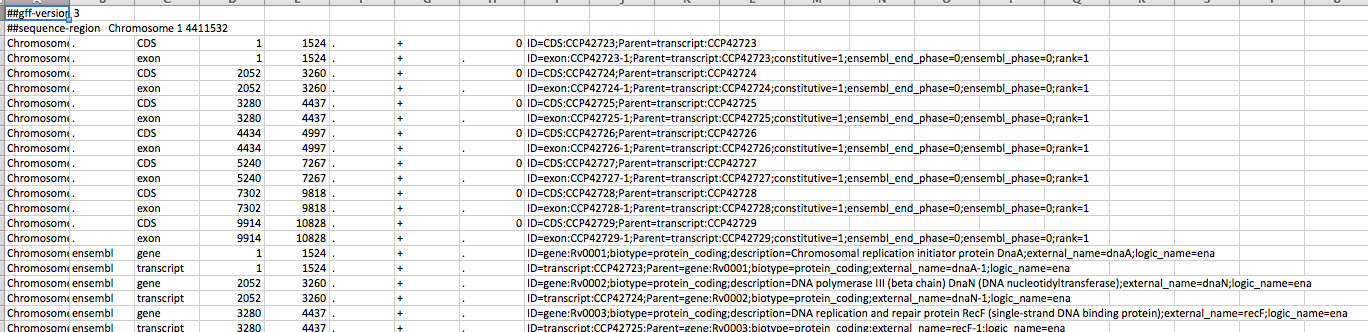


1. Sort the table and remove extraneous entries not related to genes. In this example, ‘CDS’, ‘exon’ and ‘transcript’ rows will be removed, leaving the ‘gene’ entries (red box).
2. The ‘genome_parser’ scripts were written to recognize the designation ‘CDS’ for genes. Replace the attribute category with the word ‘CDS’ for every gene.


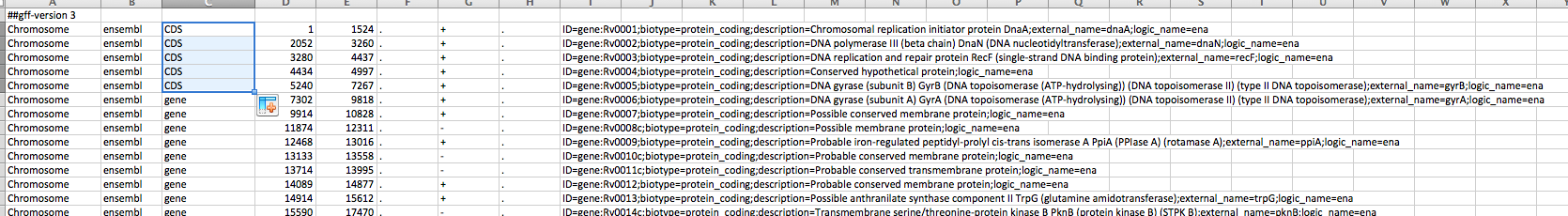


1.
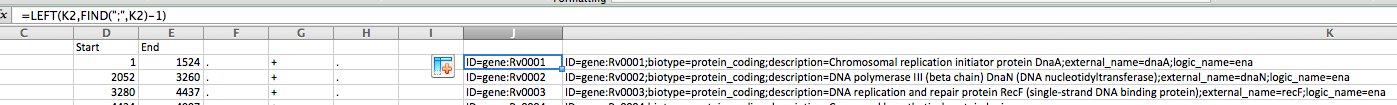
 The ‘Name’ field for the GTF file must have each gene name in the format: gene_id “geneA”. To achieve this replacement, we use the find function to define the position of the semicolon with the FIND function in Excel, and then take all the characters to the left of this using the LEFT function. Note: we use the FIND function rather than just taking a set number of characters, since it’s not guaranteed that each gene name is of the same length (e.g., in *M. tuberculosis*, some gene numbers have a ‘c’ on the end to signify being encoded on the complementary strand).
2. Copy the new column of gene names and use ‘Paste Special’ to replace all the formulas with the text values. This is required for using the replace function next.


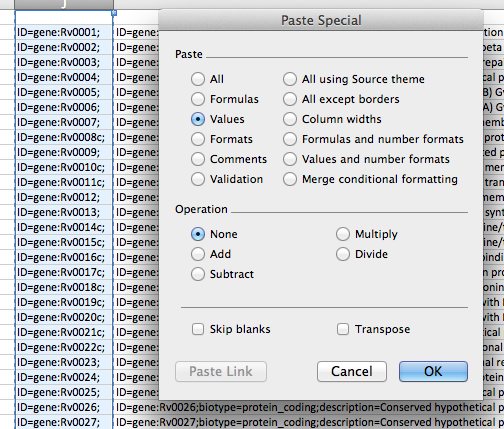


1. Using ‘replace’, find every instance of the text before the gene name (in this example, ‘ID=gene:’ and replace it with ‘gene_id “ ‘.


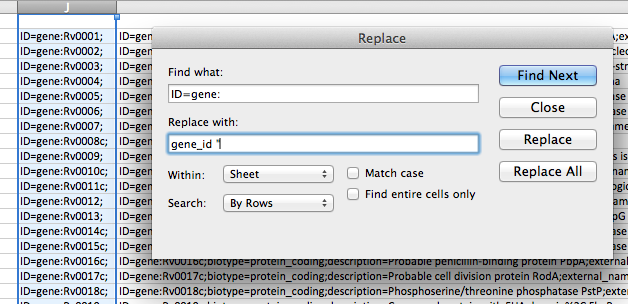


1. Next, add the last quotation mark to the end of the gene name—this will now allow the search function in the genome_parser script to pull out all the gene names in the table. You can use the CONCATENATE function to add the quotation marks. Note: In Excel, since characters must be placed between quotation marks in the formula, to add a quotation mark as a character, you must use two quotation marks in a row in the formula.


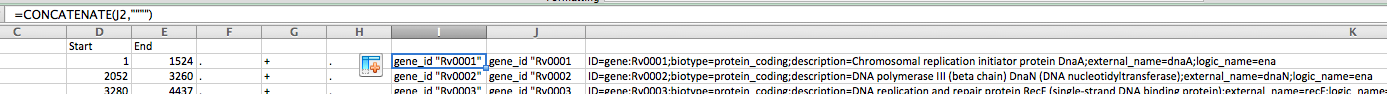


1. Rename the relevant headers in the file and then save the new file as an Excel (.xlsx) document for importing into Matlab. Note: do not convert this table to a text file format, as Excel converts the quotation marks into double quotation marks in text format.


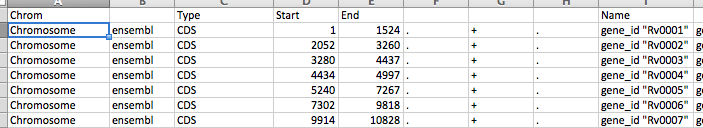


1. Import the Excel ‘GTF’ file into Matlab as a ‘table’ or ‘dataset’ (see Section 1). The relevant columns should be labeled as specified in Section 1. Unlike importing a text file, the columns with characters will all be called ‘Cell’ instead of ‘Text’, while the start and end sites will be in ‘Number’ format. Once imported into Matlab, this ‘GTF’ table is now compatible with downstream scripts.


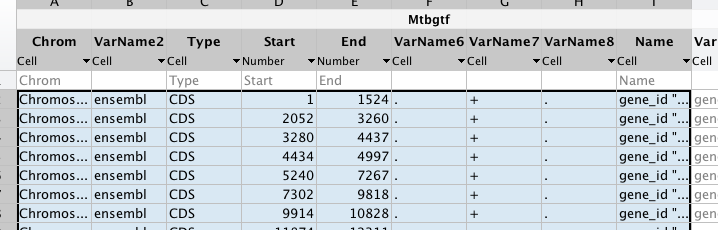


# B. Using other programs to create ARTIST compatible data

The genome_parse and SAMreader scripts provided with ARTIST are meant to help transform mapped reads into ARTIST compatible data in Matlab. However, users are welcome to use other programming methods to generate compatible data that can be fed into the ARTIST pipeline. Once you create compatible data, you can either use the Matlab import tool (see Section 1), or cut and paste from an open Excel spreadsheet (see below) to bring it into the ARTIST pipeline.

If you are pasting Excel data into Matlab, you will first create empty matrices.

Type:

Control = [];

Experiment =[];

Double click the Control and Experiment variables in the workspace (this will open a spreadsheet in the command window) and then paste in your Excel data.

For loci names, you want to use a cell array format (curly brackets), which accommodates characters, instead a typical matrix above (square brackets), which handles only numbers.

Type:

TAid={};

Double click TAid and then paste in your loci names. Note: pasting long lists of characters (e.g., loci names) may take a while (e.g., 30 min) depending on available memory.

The minimum data requirements for (Con)-ARTIST to run are shown below, and are largely similar between mariner-based and Tn5-derived TIS libraries. See the User_manual_example.mat file for example data.

**Mariner-based TIS data**


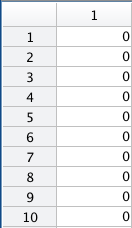

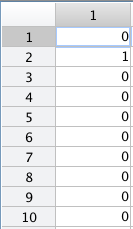

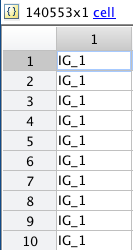


**TA sites**

**TAid**

**Control**

**Experiment**


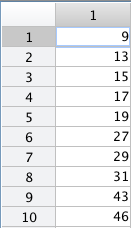


For mariner-based TIS libraries, the position of all potential insertion sites in the genome (TA sites) should be mapped—this data will be put into a matrix called ‘TAsites’. Each of these potential insertion sites should then be matched to a specific locus in the genome (this should include intergenic regions, which you may have to arbitrarily create). This list of loci is the equivalent of the TAid matrix discussed above.

Finally, from a mapping file (e.g., SAM output), you will then count all the reads from insertions that originate from each of the potential insertion sites. This creates a list of reads, where every cell lines up with its TA site position and locus ID (TAid).

From here, you will associate each insertion site to annotated genomic loci using the ‘getuniquetanames’ script (Section 5, step 2, pg. 20) to create the ‘uniquenames’ and ‘uniqueindices’ variables.

Finally, you will now proceed straight to ARTIST analysis (Sections 6 and 7).


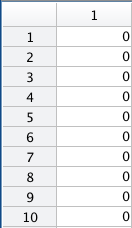

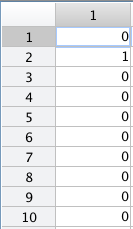

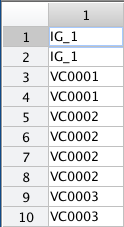

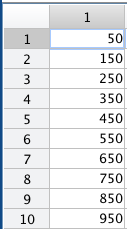
**Tn5-based TIS data**

**Control**

**Experiment**

**Tn5_annotation (aka TAid)**

**Tn5_NT (aka TAsites)**

For Tn5-based TIS data, you will need to create genomic windows of a certain size. Then you will assign these windows to a specific base position (e.g., to the midpoint position in the window); this list of window coordinates will be the equivalent of Tn5_NT (or TAsites). The Tn5_NT positions should then be mapped to a locus in the genome (including intergenic regions); this list of loci is in the same format as in the Tn5_annotations matrix (equivalent to TAid).

Next, you will add all the reads that map into each window and assign these reads to your Control or Experiment arrays. From here, you will associate each insertion site to annotated genomic loci using the ‘getuniquetanames’ script (Section 5, step 2, pg. 19) to create the ‘uniquenames’ and ‘uniqueindices’ variables.

Finally, you will now proceed directly to the ARTIST pipeline (Sections 6 and 7).

# C. Running Con-ARTIST with resampling and MWU but without HMM

Con-ARTIST was created to analyze consistent changes in read counts as a single library is grown in different conditions. As such, we have found that HMM analysis of independently generated TIS libraries was problematic, since transposons may insert at different sites in independently generated libraries, even within the same genes. This heterogeneity of insertion sites between libraries makes it difficult for the HMM to consistently define a unidirectional shift in reads between conditions, and we have seen Con-ARTIST produce more spurious results on this type of data than straightforward hypothesis-based testing (e.g., MWU tests alone). Therefore, we recommend using aggregative statistical approaches such as the MWU test over HMM in these cases to assess whether reads in a given locus are significantly altered under different conditions. The drawback in this analysis is that MWU tests are limited to annotated loci and will not define domains within genes that may be required for growth in different conditions. In preliminary work, this methodology appears to produce accurate and statistically rigorous results (data not shown), but the results will be restricted to annotated loci rather than providing high-resolution insight at the insertion level.

1. Carry out Con-ARTIST’s simulation-based normalization function to reduce interference from stochastic changes in mutant populations the simulation (see Section 7 for details).

Type:

[Controlsims] = simulateequalsaturation (length(Experiment(Experiment~=0))/length(Control(Control~=0)), sum(Experiment), Control, 100);

The length(Experiment(Experiment~=0))/length(Control(Control~=0)) input gives the relative proportion of TA sites disrupted in the experimental library versus the control.

The sum(Experiment) command gives the total number of reads to simulate.

100 is the number of simulations to run. You may run more if you like—we found that 1000 was no different than 100 in our data, and the drawback with more simulations is lengthier computation time downstream.

Note: Since we are comparing two datasets, either both the Control and Experimental libraries should be normalized for replication bias (Section D1), or both libraries should be the uncorrected raw data. We used raw data for our analyses here.

Also, since these libraries are independently generated, the Experimental library may contain more unique insertions than the Control library. In this instance, you will carry out the script above but flip the positions of Control and Experiment. This will not affect the MWU tests below, but the count ratio created in step 3 will be flipped (large ratio indicated conditionally essential locus, while small ratio signifies and enriched locus).

1. Carry out independent MWU analyses on all loci. This script may take upwards of 30 minutes to run, depending on the number of loci to be compared and number of control simulations previously performed.

Type:

[MWUboots] = runmwuallboots (Controlsims, Experiment, uniquenames, uniqueindices);

This script uses each of the Controlsims to compare against the Experiment data with a MWU statistical test. Every row in the MWUboots output file corresponds to the same cell in the uniquenames matrix. The number of columns in MWUboots depends on the number of simulations carried out previously (e.g., 100 simulations = 100 independent MWU tests). Each cell contains the p-value from carrying out a MWU test on the Control simulation and Experimental data for that locus.

1. Report the average p-values and reproducibility of significance from the MWU tests for every locus.

Type:

[MWUstats]=MWUsummarystats(MWUboots, 0.001, 0.9, uniqueindices, Controlsims, Experiment);

This script will take the MWU p-values from the MWUboots array (created above) and average them across all 100 MWU tests.

The script will also assess the fraction of those tests that produced a p-value lower than the cutoff specified in the input (e.g., 0.001), and whether the fraction of significant simulations is greater than the reproducibility threshold you set (e.g., 0.9).

Finally, you will input the uniqueindices, Controlsims and Experiment arrays for the script to calculate the ratio of reads per locus between conditions.

In the example above, we are asking how many MWU tests for each locus had a p-value less than 0.0001. Also, whether the locus produced a p-value of < 0.001 in over 90% of the MWU tests.

The output matrix, MWUstats, will contain 6 columns with the same number (and order) of rows as the uniquenames loci.

Column 1 is the proportion of MWU tests that the locus was called significant (i.e., p-value was less than the one specified by the user).

Column 2 is whether this locus was reproducibly significant in more tests than the user cutoff (1 = yes; 0 = no).

Column 3 is the average p-value for each locus across all MWU simulations.

Column 4 is the standard deviation in p-values for each locus across all MWU simulations.

Column 5 is the average read count ratio from all insertions within each locus between the experimental and control dataset across 100 simulations. The ratio is effectively (Experiment reads/Controlsim reads); large ratios denote enriched loci, while low ratios indicate conditional essentiality. Note: the script performs some adjustment for loci that might otherwise give mathematical errors in Matlab. For loci that have no reads in the Experiment dataset, but reads in the Controlsim dataset, we arbitrarily set the Experiment read to 1, so that we can caulate a ratio. Conversely, if there are reads in Experiment, but not Controlsim, we set Controlsim reads to 1 to avoid a mathematical error. Finally, if there are no reads in either the Experiment or Controlsim library (i.e., the locus appear essential for growth in both conditions), the ratio reported is ‘0’.

Column 6 is the standard deviation of read counts for each locus across the 100 different simulations.

1. You can copy and paste the MWUstats matrix next to the uniquenames list of loci in a spreadsheet for easier downstream curating.

# D. Output ARTIST results in other data formats

The easiest way to access and save the ARTIST data for spreadsheet analysis is to directly open each matrix in the command window, copy that data and paste it into Excel spreadsheets. However, it may be useful to export the matrices as smaller files for quick transfer between machines.

Comma-separated value (csv) format is a standard text format that minimizes file space and can be opened in a variety of programs. To export specific files in csv format, you can use Matlab’s ‘csvwrite’ function (below). Note: Matlab also contains an ‘xlswrite’ function that is meant to create Excel readable spreadsheets. However, we have had difficulty with using this function as different versions of Matlab may or may not communicate well with different versions of Excel.

Type:

csvwrite(‘output_file’,matrix_name);

In the example above, the name of output file is in quotes, no file extension necessary. The input is the name of the matrix on your workspace, no quotes. The output file will be created in your current working folder.

# References

1. Chao, M.C., Pritchard, J.R., Zhang, Y.J., Rubin, E.J., Livny, J., Davis, B.M. and Waldor, M.K. (2013) High-resolution definition of the Vibrio cholerae essential gene set with hidden Markov model-based analyses of transposon-insertion sequencing data. *Nucleic Acids Res*, **41**, 9033-9048.

2. Zhang, Y.J., Ioerger, T.R., Huttenhower, C., Long, J.E., Sassetti, C.M., Sacchettini, J.C. and Rubin, E.J. (2012) Global assessment of genomic regions required for growth in Mycobacterium tuberculosis. *PLoS Pathog*, **8**, e1002946.

3. Gallagher, L.A., Shendure, J. and Manoil, C. (2011) Genome-scale identification of resistance functions in Pseudomonas aeruginosa using Tn-seq. *MBio*, **2**, e00315-00310.

4. Zhang, Y.J., Reddy, M.C., Ioerger, T.R., Rothchild, A.C., Dartois, V., Schuster, B.M., Trauner, A., Wallis, D., Galaviz, S., Huttenhower, C. *et al.* (2013) Tryptophan biosynthesis protects mycobacteria from CD4 T-cell-mediated killing. *Cell*, **155**, 1296-1308.
